# The prevalence and severity of loneliness and deficits in perceived social support among who have received a ‘personality disorder’ diagnosis or have relevant traits: a systematic review

**DOI:** 10.1186/s12888-023-05471-8

**Published:** 2024-01-03

**Authors:** Sarah Ikhtabi, Alexandra Pitman, Lucy Maconick, Eiluned Pearce, Oliver Dale, Sarah Rowe, Sonia Johnson

**Affiliations:** 1 UCL Division of Psychiatry, London, UK; 2 UCL Division of Psychiatry, Camden and Islington NHS Foundation Trust, London, UK; 3 UCL Division of Psychiatry, NIHR Doctoral Research Fellow, Camden and Islington NHS Foundation Trust, London, UK; 4 Sussex Partnership Foundation Trust, London, UK

**Keywords:** Loneliness, Perceived social support, Personality disorder, Complex emotional needs, Systematic review, Literature review

## Abstract

**Background:**

Loneliness and struggles with unmet social needs are a common experience among people with ‘personality disorder’ diagnoses/traits. Given the impact of loneliness and poor perceived social support on mental health, and the importance of a sense of belonging for recovery, a systematic review examining the prevalence/severity of loneliness and deficits in perceived social support among people with ‘personality disorder’ diagnoses/traits is an essential step towards developing an intervention targeting the social needs of people with diagnoses/traits ‘personality disorder’. Despite an extensive literature on loneliness and deficits of perceived social support among people with ‘personality disorder’ diagnosis/traits, to date there has been no systematic review of this evidence.

**Method:**

We conducted a systematic review synthesising quantitative data on the prevalence/severity of loneliness and deficits of perceived social support among people with diagnoses/traits of ‘personality disorder’ in comparison with other clinical groups and the general population. We searched Medline, Embase, PsycINFO, Web of Social Science, Google scholar and Ethos British Library from inception to December 2021. We conducted quality appraisals using the Joanna Briggs Critical appraisal tools and rated the certainty of evidence using the Grading of Recommendation, Assessment, Development and Evaluation approach. A narrative synthesis was used describing the direction and strength of associations prioritising high quality studies.

**Findings:**

A final set of 70 studies are included in this review, most of which are cross-sectional studies *(n = 55),* based in the United States *(51%)* and focused on community samples. Our synthesis of evidence found that, across all types of ‘personality disorders’ (except ‘narcissistic personality’ traits), people with traits associated with ‘personality disorder’ or meeting criteria for a diagnosis of ‘personality disorder’, have higher levels of loneliness, lower perceived relationship satisfaction, and poorer social support than the general population or other clinical samples.

**Conclusion:**

The quality of evidence is judged as low quality. However, given the distressing nature of loneliness and the known negative effects of loneliness on mental health and recovery, it is important for future research to explore mechanisms by which loneliness may exacerbate ‘personality disorder’ symptoms and the impact this has on recovery.

**Supplementary Information:**

The online version contains supplementary material available at 10.1186/s12888-023-05471-8.

## Introduction

There is an increasing interest in the social factors associated with mental ill health among people with ‘personality disorder’ diagnoses/traits, both as protective factors and barriers to recovery. Our recent meta-synthesis of the qualitative literature on experiences of loneliness among people with ‘personality disorder’ diagnoses/traits has described experiences of an intense sense of disconnection and struggle with unmet social needs [1]. However, there is little quantitative evidence assessing loneliness and perceived social support (PSS) as an outcome of interest among people with ‘personality disorder’ diagnoses/traits [2]. Despite loneliness and PSS being potentially important intervention targets, these social factors are overlooked as a possible target for interventions [2]. Therefore, to build the groundwork required for a future co-developed social intervention and an understanding of the current state of the evidence on loneliness/PSS among people with ‘personality disorder’ diagnosis/traits, a systematic review on the prevalence/severity of loneliness and potential deficits in/lack of PSS among people with ‘personality disorder’ diagnoses/traits is timely and necessary.

### Loneliness and perceived social support (PSS)

Loneliness is a painful, subjective, emotional experience characterised by a perceived discrepancy between actual and desired patterns of social interaction [3, 4]. Loneliness is a sign of unfulfilling relationships and is a possible indicator of interpersonal problems and impoverished social relationships that interfere with a person’s sense of belonging [5]. Loneliness is associated with social network size only weakly or moderately [3]. According to Weiss’s typology, loneliness is a multidimensional phenomenon categorized into different forms of loneliness-related experiences such as emotional loneliness and social loneliness [6]. Emotional loneliness arises from a lack of close and intimate emotional attachment [6]. Social loneliness occurs when there is a lack of or restricted social network [6]. Another facet of social relationships is PSS, which like loneliness, is subjective and compromises one’s perceptions of the social world [7]. PSS refers to a person’s beliefs regarding the adequacy of their social resources available, and research indicates that PSS has a significant impact on mental health outcomes [7]. The literature exploring loneliness and PSS indicates that PSS is negatively associated with levels of loneliness and is an important variable that predicts, protects against, and reduces levels of loneliness [7–9]. Deficiencies in PSS and loneliness are linked to a wide range of mental and physical health problems and increase the risk for many mental health problems [8–10]. Therefore, PSS and loneliness may both influence recovery outcomes in people with ‘personality disorder’ diagnoses/traits, requiring investigation of each as an important target for therapeutic intervention.

### Ongoing debate: ‘personality disorder’

There is ongoing debate regarding the diagnostic label ‘personality disorder’, which has been criticized as being stigmatizing [11]. Arguments against it include that it implies a defective personality and places blame on the individuals themselves whilst underestimating the potential role that a history of complex trauma or difficulties relating to others play in the development of the associated symptoms [11–14]. The issues associated with a categorical diagnosis of ‘personality disorder’ are further problematic as there is a lack of robust evidence supporting the 10 categories of ‘personality disorder’ and issues of low reliability [11]. More recently, some people diagnosable with a ‘personality disorder’ prefer alternative descriptions such as the term ‘complex emotional needs’ (CEN) [13, 15]. CEN is also used as a broad term to include people who may have ‘personality disorder’ traits. We promote and support co-produced efforts to develop other preferable alternative and better ways of describing the needs of people who have symptoms that align with the criteria of ‘personality disorder’. As there is no agreed upon acceptable term, and given the predominant use of the term ‘personality disorder’ in academic research, we employ the term ‘personality disorder’ as an umbrella term to address the needs of people who are diagnosed with ‘personality disorder’, or people with traits of ‘personality disorder’ based on dimensional assessments of ‘personality disorder’ symptoms or traits through self- or clinician- or researcher-assessment. We use quotation marks to demonstrate that we believe this term requires further review.

### Loneliness, perceived social support and ‘personality disorder’

People with ‘personality disorder’ diagnoses/traits are particularly vulnerable to difficulties with forming and maintaining satisfying social connections, as they report interpersonal problems and difficulties managing social relationships [16–19]. A quantitative cross-sectional study describing the intensity of loneliness in the lives of people with a diagnosis of ‘emotionally unstable personality disorder’ (‘EUPD’) found that low social functioning and objective social isolation did not account for the severity of loneliness experienced by people with ‘EUPD’ [20]. These findings illustrate that factors beyond objective social isolation, social network features and social functioning contribute to the intense loneliness experienced.

Our meta-synthesis of the qualitative literature describing experiences of loneliness in people with ‘personality disorder’ diagnoses/traits further support the notion that the prevalence and severity of loneliness are often associated with factors other than objective social concepts such as social network size [1]. It appears that the intense feelings of loneliness experienced by people with ‘personality disorder’ diagnoses/traits are often perceived as associated with traumatic experiences of alienation and rejection during childhood, which can also continue into adulthood [1]. These early experiences and maladaptive cognitive processes may also be intensified by discriminatory experiences and stigma that further exacerbate feelings of loneliness [21, 22]. In keeping with these qualitative reports, another British cross-sectional study exploring loneliness in people with a range of psychiatric diagnoses found that, among people diagnosed with ‘personality disorder’, loneliness is associated with higher rates of perceived and internalized discrimination [22]. Collectively, quantitative and qualitative findings point to the complex nature of loneliness and the need to further explore prevalence/severity of loneliness and the deficits of PSS among people with ‘personality disorder’ diagnoses/traits.

Despite the calls for a focus on social needs, loneliness research in the context of ‘personality disorder’ is overlooked as a possible target for interventions, with very few studies assessing loneliness as a primary outcome of interest [2] No systematic review to date has been conducted exploring the prevalence/severity of loneliness and deficits in PSS among people with ‘personality disorder’ diagnoses/traits. Although loneliness interventions have been developed and have undergone preliminary evaluations for a group of people with overlapping difficulties such as complex and severe depression and anxiety [23], we still need interventions that address the social needs of people with ‘personality disorder’ diagnoses/traits. Challenges described by people with ‘personality disorder’ diagnoses/traits that should be priorities for future intervention targets include difficulties making and maintaining social connections, longing for fellowship, lack of purposeful and meaningful social activity, and feelings of otherness and alienation [1]. As an initial step towards the goal of developing broader therapeutic approaches, our aim in this systematic review is to provide a comprehensive synthesis of the current evidence on the prevalence and severity of loneliness and deficits of PSS among people with ‘personality disorder’ diagnoses/traits, comparing this to other clinical groups and the general population.

## Method

We conducted a systematic review synthesising the quantitative research literature on the severity and/or prevalence of loneliness and deficits of PSS among people with ‘personality disorder’ diagnoses/traits. This systematic review follows PRISMA guidelines (See Supplementary file 5).

Our main research question was:

- What is the prevalence and severity of loneliness and deficits in PSS in people with ‘personality disorder’ diagnoses/traits, and how does it compare to those in other clinical groups and the general population?

This review protocol was pre-registered on PROSPERO (registration number: CRD42022321587). We have made one amendment in the protocol during title/abstract screening stage: deciding to expand our data synthesis strategy to include the potential for a meta-analysis, but otherwise tabulate data and conduct a narrative synthesis in the event of high heterogeneity.

### Search strategy

We used the following four electronic bibliographical databases to conduct a search from database inception to December 13, 2021: Medline, Embase, PsycINFO, and Web of Social Science. We conducted Google Scholar search along with an Ethos British Library database search to retrieve any dissertations or PhD theses papers on this topic that were not published in a journal.

The search terms were chosen and constructed to identify quantitative studies investigating a range of social concepts, including objective social isolation and related concepts, such as confiding relationships, categorical and dimensional approaches to ‘personality disorder’ (See Supplementary Appendix 1). The search terms identified aimed to capture data exploring conceptually overlapping terminology associated with loneliness (i.e. social isolation) to ensure comprehensive retrieval of relevant papers. We also included objective social measures to cover concepts explored in a separate systematic review of studies describing the prevalence and degree of social isolation among people with ‘personality disorder’ diagnoses/traits also conducted by members of our team. With the aim of identifying relevant literature more comprehensively, we included search terms such as ‘complex emotional needs’ that have been used by authors wishing to avoid the stigma associated with the ‘personality disorder’ term [13], and further incorporated phrases that have been used to describe people who may have traits suggestive of ‘personality disorder’ [2, 13, 15]. In keeping with current practices of assessing ‘personality disorder’, we included categorical and dimensional approaches to assessing the symptoms and severity of ‘personality disorder’ in clinical and general population samples. Examples of dimensional assessment measures include Standardised Assessment of Personality Abbreviated Scale (SAPAS) [24] and Narcissistic Personality Inventory [25].

We built on a set of comprehensive and inclusive search terms capturing the concepts of loneliness and ‘personality disorder’ that had been developed and employed in two previous meta-syntheses by our team [1, 4], and a conceptual review by Wang and colleagues [26]. If studies were unclear as to eligibility or data, and/or requests for full texts were required, we emailed the authors of the paper. A four-week period was allowed for a reply before excluding the article based on insufficient information.

### Inclusion criteria and exclusion criteria

We included epidemiological studies that reported primary data describing 1) the estimated point or period prevalence, and/or severity of loneliness in people with ‘personality disorder’ diagnoses/traits and 2) deficits in or lack of PSS. We also included studies that compared people with ‘personality disorder’ diagnoses/traits to other clinical groups or the general population (See Supplementary Table 1 for Inclusion and Exclusion criteria). We included studies using any type of validated self-report measure of loneliness or PSS, such as the UCLA Loneliness Scale [27] or Multidimensional Scale of Perceived Social Support [28].

### Outcomes

The main outcomes of interest were prevalence and severity of loneliness and deficits in PSS among people with ‘personality disorder’ diagnoses/traits. We aimed to derive overall estimates and compare these to the prevalence/severity of loneliness and PSS in other clinical groups and in the general population. We included studies using measures of loneliness with evidence supporting reliability and/or validity, such as the Social Network Analysis Likert scale for assessing perceived social network quality [29]. For comprehensiveness, we also included studies assessing perceptions of the quality of social networks with evidence supporting reliability and/or validity.

### Data screening, data extraction, and quality assessment

After deduplication, the primary researcher (SI) independently screened the titles and abstracts of potentially relevant articles against the inclusion criteria to assess eligibility. Another researcher (LM) independently screened a randomly selected 10% of titles and abstracts to check for adherence with the criteria and agreement. SI then conducted a full-text screen to establish a final set of eligible articles. LM independently screened the full texts of a randomly selected 10% of articles, checking for agreements on eligibility and discussing any disagreements with SI. Any further disagreements or queries regarding the inclusion criteria were resolved by discussion with an experienced third reviewer (SJ).

A standardized data extraction form was developed and employed to gather relevant information on study characteristics and key relevant findings (See Supplementary Tables 2-9). Two review authors (SI and LM) independently assessed the methodological quality of each article. The second reviewer (LM) conducted a quality appraisal for 10% of the total articles. Any disagreements were resolved through discussion at the end of the data-extraction process. Disagreements between the two reviewers were resolved through a discussion with a third reviewer (SJ).

To assess the methodological quality of cohort studies on loneliness or PSS and ‘personality disorder’, we used the Joanna Briggs Institute Cohort Critical Appraisal tool, whilst for cross-sectional studies we used the Joanna Briggs Institute Cross-sectional Critical Appraisal tool [30]. These tools evaluate to what extent the included study addressed issues associated with bias in design, conduct and analysis. Studies are categorised by risk of bias into low risk or high risk. We also rated the certainty of the evidence using the GRADE (Grading of Recommendation, Assessment, Development and Evaluation) approach in relation to our two outcomes (See Supplementary Table 10). This approach is used in the absence of a single estimate of effect or meta-analysis and is based on the following domains: study design, risk of bias, inconsistency of evidence, indirectness of the evidence to the main research question(s), imprecision of estimates, and potential publication bias [31, 32]. These domains contribute to a final rating of the certainty of evidence regarding each outcome of interest, categorised as very low, low, medium or high [31, 32]. The level of certainty can be rated up where there is a large magnitude of effect, and/or a dose-response relationship, or where there is likely to be residual confounding [32]. In absence of a single effect estimate, we used the GRADE criterion described by Murad et al. which has been increasingly used in narrative synthesis [32].

### Data synthesis

We conducted a narrative synthesis of results focusing on the prevalence or/and severity of loneliness and deficits in PSS and comparing these to other clinical groups and the general population. We used Campbell et al’s [33] and Rodgers et al’s [34] methodological guidance on reporting narrative synthesis to promote transparency and in the case of heterogeneity in the direction of findings (e.g. negative and positive association between loneliness and ‘personality disorder’ presented) [33]. We described the methods used (whether cross-sectional or longitudinal) and the strength and direction of the association between these subjective social factors and ‘personality disorder’, exploring how loneliness and PSS differed by the type of ‘personality disorder’.

In our summary we give more prominence to the findings of the 53 studies that we rated as high-quality (scoring at least a 6/8 for cross-sectional studies or 9/11 for longitudinal studies using respective Joanna Briggs tools) in our narrative synthesis. However, we also report findings from the lower quality studies where that was necessary in expanding upon findings. These studies rated as lower quality are included either at the end of a sub-section where needed to qualify findings from studies rated as high-quality and described as ‘additional studies rated as low-quality’ to distinguish them.

## Results

**Fig. 1:**
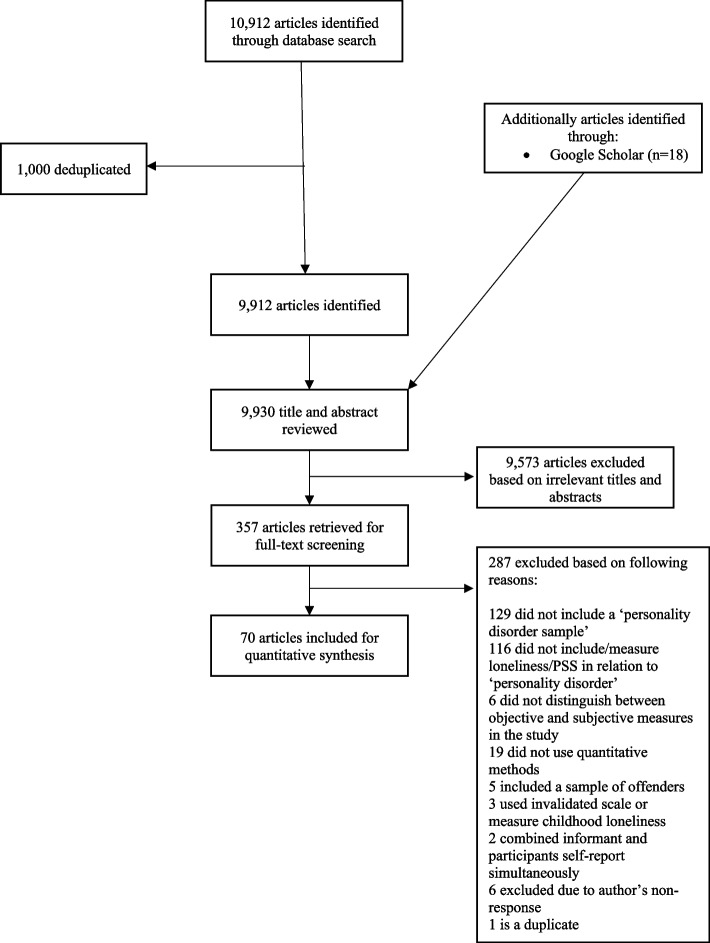
Flow diagram of studies through systematic review process

A total of 10,912 citations were retrieved through the database (See Fig. 1 for study selection process). A total of 9573 articles were excluded after title/abstract screening process, leaving a total of 357 articles for full-text screening. A final set of 70 articles meeting the inclusion criteria were included. Inter-rater reliability was high, we achieved 95 and 79% agreement of the title/abstract and full-text screening, respectively; and through discussion between two independent screeners (LM and SI), we achieved 100% agreement on decisions to include/exclude articles.

Characteristics and quality of all included studies are described in Supplementary Tables S2-9. Most studies (75%) were rated as low risk of bias; however, studies often did not identify confounders and/or use strategies to deal with confounders. For both outcomes (loneliness and PSS), we judged the certainty of evidence to be low (See Supplementary Table 10). In total 33,160 participants were included, with sample sizes ranging from 22 to 11,329. Most studies were cross-sectional (*n* = 55), including five cross-sectional social network analysis studies [29, 35–39], and 15 were longitudinal, including four longitudinal social network analysis studies [40–43]. Twenty studies had a comparison group, either from the general population (*n* = 13) or with other psychiatric disorders (*n* = 7). The majority of studies were set in the United States (US: *n* = 35; 51%); other settings were the United Kingdom (UK), Continental Europe, Canada, Norway, Australia, China, Japan, Turkey, and Israel.

Majority of participants in most studies were women and ages ranged from 12 to 99 years, with two studies focusing on adolescents aged 12 to 19 years [24, 44]. Many studies focused exclusively on community samples of people with traits/a diagnosis of ‘emotionally unstable personality disorder’ or traits of ‘narcissistic personality’ and assessed these traits using self-reported measures such as McLean Screening Instrument for Borderline Personality Disorder [45] and The Narcissistic Personality Inventory [25].

In presenting our results we structured findings by each type of ‘personality disorder’, for loneliness and PSS separately, combining findings in relation to ‘dependent personality disorder’ and ‘avoidant personality disorder’ in the sub-sections covering mixed samples of people with ‘personality disorder’ due to the small number of studies in those categories.

### Loneliness and other subjective social measures among people with diagnoses/traits of ‘personality disorder’ (mixed samples)

Eleven studies, nine of which are cross-sectional studies, examined subjective social factors among mixed samples of people with a range of ‘personality disorder’ diagnoses/traits, and demonstrated a positive association between loneliness, or similar measures, and severity of symptoms associated with a ‘personality disorder’ [22, 46–52] (See Supplementary Table 2). Studies found that people with a diagnosis of ‘personality disorder’ reported higher levels of dissatisfaction with their social relationships and loneliness compared to people with psychosis and common mental health disorders (CMD) and with the general population [22, 47, 51]. Of the nine studies rated as high-quality, Abrams and colleagues (1996) demonstrated that, among a U.S sample aged 60-85 years, some trait scores of Cluster B and C disorders were inversely correlated with the presence of satisfying relationships [50]. Correlation coefficients for traits ranged from -0.348 for ‘paranoid personality disorder’ to -0.501 for ‘schizoid personality disorder’ (*P*<0.05). Alasmawi and colleagues (22) conducted a cross-sectional study of a British sample of people with a primary diagnosis of psychotic disorders (n = 106), CMDs (n = 49), and ‘personality disorders’ (*n* = 37) (22). This study found that people with a ‘personality disorder’ diagnosis experienced the highest level of loneliness compared to people with CMDs and psychosis, after adjusting for social and psychological factors including perceived discrimination [22]. Another cross-sectional Swiss study rated high-quality also reported that ‘EUPD’ was significantly associated to feeling frequently lonely compared to other ‘personality disorder’ traits such as those of ‘histrionic personality disorder’ [48].

Six studies, five of which were U.S based and one Swiss study, investigated loneliness among people with ‘Cluster C personality disorders’ or traits [48, 49, 52–55]. One study rated as high-quality indicated that people with a diagnosis of ‘AVPD’ scored lower on perceptions of belonging than those with an ‘EUPD’ diagnosis and control group on perceptions of belongingness [52]. A cross-sectional Swiss study, rated as high-quality, indicated that ‘dependent personality disorder’ traits were significantly associated to frequently feeling lonely (frequently lonely: B = 0.256 (SE = 0.081), *p* = 0.002) [48]. An additional longitudinal study rated as low-quality reported that participants scoring higher on the ‘dependence personality’ style scale show higher loneliness at time 1 and at time 2 (10 weeks later) [55]. Subjects scoring higher on the dependency scale showed consistently higher loneliness [55].

### PSS among people with ‘personality disorder’ diagnoses/traits (mixed samples)

Ten studies, five of which are cross-sectional studies, investigated PSS among people with a range of different diagnoses/traits of ‘personality disorder’ (See Supplementary Table 3). Two studies indicated that the majority of their sample (67.2 and 68%) had a diagnosis of ‘personality disorder’ [56, 57].

All 10 studies showed that a diagnosis/traits of ‘personality disorder’ was associated with lower PSS compared to people without a diagnosis/traits of ‘personality disorder’ [24, 56–65]. A large cross-sectional survey of a UK-based psychiatric sample, rated as high-quality, found that having high levels of emotional support is associated with decreased odds of ‘personality difficulties’, as assessed using the Standardised Assessment of Personality—Abbreviated Scale (Adjusted odds ratio = 0.41 (0.25-0.66 95% CI) *p* ≤ 0.001) controlling for a variety of sociodemographic factors and social network size [62]. A Finnish study rated as high-quality similarly indicated that more social support from close friends is associated with fewer symptoms of ‘AVPD’ and ‘schizoid personality disorder’ traits [24]. A U.S. study rated as high-quality reported a significant inverse correlation between personality disorder and perceived support quality with coefficients ranging from - 0.03 for people with a diagnosis of ‘dependent personality disorder’(DPD) and -0.23 (p ≤.01) for people with ‘AVPD’ [61]. Two studies sampling U.S and Norwegian subjects, rated as high-quality, assessed PSS among people with a diagnosis of ‘AVPD’ and social anxiety disorder (SAD) [59, 60]. These studies found that people with an ‘AVPD’ diagnosis or people who endorsed more ‘AVPD’ traits reported lower PSS compared to those with SAD [59, 60]. Another multi-site Norwegian study rated as low-quality included 1023 patients with ‘personality disorder’ with the majority largely (40%) diagnosed with ‘AVPD (64). This study indicated that both ‘AVPD’ and ‘EUPD ‘diagnoses were correlated with less social support than in general population controls, with poorer PSS for ‘AVPD’ than ‘EUPD’.

### Loneliness and other subjective social measures among people with ‘narcissistic personality’ traits

Thirteen cross-sectional studies investigated the relationship between loneliness and ‘narcissistic personality’ traits in community/general population samples [25, 35, 46, 66–75] such as a sample of undergraduate students [69]. All but four of the studies reported a significant positive relationship between narcissistic traits and loneliness (See Supplementary Table 4) [25, 68, 71, 74]. According to three cross-sectional studies, a Canadian study rated as high-quality and a US and a Polish study rated as low-quality, the traits termed vulnerable or covert narcissism were both moderately associated with loneliness [35, 67, 70]. Conversely, grandiose or overt narcissism was less associated with loneliness [35, 67, 70]. Additional cross-sectional studies rated as low-quality exploring narcissistic traits, as measured using the Narcissistic Personality Inventory (NPI) in a sample of Turkish, Italian, and British undergraduate students, indicated that narcissism is inversely associated with a chronic state of loneliness [25, 68, 71]. However, one social network analysis study of U.S participants, rated as low-quality, allowed for a more detailed analysis of the way an individual’s social networks were uniquely perceived by people with higher grandiose narcissism versus higher vulnerable narcissism traits [35]. This weak evidence suggested that vulnerable narcissism may be associated with feeling less closeness to others in the network and more envy, but that grandiose narcissism may be associated with perceptions of others as “self-centred” [35].

### PSS and ‘narcissistic personality’ traits

Only three cross-sectional studies explored the relationship between narcissistic traits and PSS [24, 72, 76] (See Supplementary Table 5). As with loneliness, the type of narcissistic dimensions or traits investigated can potentially influence the direction of the relationship with PSS. Although the findings on the relationship between ‘narcissistic traits’ and PSS were mixed, a high-quality longitudinal study of Finnish adolescents found that young people with greater PSS at baseline showed a greater decline in narcissistic traits over time [24]. One US study rated as high-quality found that grandiose narcissism was associated with higher levels of PSS social support, but ‘vulnerable narcissism’ with lower levels [76].

### Loneliness and other subjective social measures among people with ‘Cluster A Personality Disorder’ or traits

Seven studies measured associations between ‘schizoid’/ ‘schizotypal’ features and loneliness, all finding the association to be positive [77–83] (See Supplementary Table 6). Three studies from Israel or the US, rated as high-quality, found positive associations between ‘schizoid’ features/ schizotypal traits and loneliness [78, 79, 83], with correlation coefficients ranging from 0.39 to 0.50 (*p* < 0.001). A study indicated that people who had ‘medically serious’ suicide attempts endorsed more schizoid features and reported higher levels of loneliness [79]. The only longitudinal study in this category, rated as high-quality, analysed three waves of network analysis data collected during COVID-19 among subjects from UK, USA, Greece, and Italy. They found a strong association between the negative dimension of ‘schizotypal’ traits, particularly interpersonal deficits, and loneliness (r = 0.619) [82]. A study of Norwegian undergraduate students, rated as high-quality, examined the relationship between loneliness, ‘schizotypal’ symptoms, and psychotic-like symptoms, finding that higher levels of loneliness were significantly and positively associated with positive ‘schizotypy’ traits [83].

Two cross-sectional studies investigated the relationship between loneliness and ‘schizotypal’ symptoms among students in the general population. Findings from the Australian sample [77] and from US sample [80], both rated as low-quality, were similar in finding a positive association between negative ‘schizotypal’ symptoms and loneliness (*r* = .51 - .60). Negative ‘schizotypal’ is characterised by social anxiety, anhedonia, diminished positive affect [77, 80].

### PSS and ‘Cluster A Personality Disorder’ or traits

Four cross-sectional design studies, all rated as high-quality, assessed the relationship between ‘cluster A personality disorder’ or traits, such as ‘schizoid/schizotypal personality disorder’ and ‘paranoid personality disorder’, and PSS (See Supplementary Table 7). PSS as measured by the interpersonal support evaluation list (ISEL) and the social support questionnaire was found to be negatively correlated with ‘schizoid/schizotypal’ and ‘paranoid personality disorder’, with correlation coefficients ranging from -.37 to -.50 (*p* < .005), in one study [84], and –.18 to -.29, in another study [85]. A U.S study rated as high-quality found that people with high schizotypy perceived less social support than people with low schizotypy [86].

There were some studies that indicated differential correlational findings regarding which type of ‘cluster A personality disorders’ traits/diagnosis has the strongest association with PSS. One U.S study rated as high-quality that extracted baseline findings from a longitudinal study of people scoring high on social anhedonia and what they termed “demographically matched non-anhedonic participants” found a significant negative association between ‘schizoid/schizotypal personality disorder ‘and ‘paranoid personality disorder’, and PSS, with the strongest association between ‘schizoid personality disorder’ and PSS [84]. Conversely, another U.S study rated as high-quality investigated the association between ‘cluster A personality disorder’ and PSS among people with high social anhedonia and found that, out of all ‘Cluster A personality disorders’, the magnitude of the association is strongest between ‘paranoid personality disorder’ and PSS [85].

### Loneliness and other subjective social measures among people with a diagnosis or traits of ‘Emotionally Unstable Personality Disorder’ (EUPD)

All 16 studies, 11 of which are cross-sectional studies, in this category found either higher levels of loneliness among people with ‘EUPD’, compared to people with depression and psychosis, or significant positive associations between low perceived relationship satisfaction/ higher loneliness and ‘EUPD’. The evidence also suggested that levels of loneliness are associated with individual symptoms of ‘EUPD’, such as identity disturbances and self-harm (See Supplementary Table 8). Based on these studies, ‘EUPD’ traits were found to be significantly and positively associated with loneliness [44, 87–91]. Three studies, 2 U.S and one German, all rated as high-quality apart from one German study rated as low-quality, found that levels of loneliness and relationship quality were significantly associated with ‘emotionally unstable personality’ symptoms even when other relevant social and psychological variables were controlled for, such as trauma and baseline perceptions of relationship quality [40, 87, 88]. One US longitudinal study, rated as high-quality, defined social isolation as “having no emotionally sustaining relationships outside of the family” and found that patients diagnosed with ‘EUPD’ were significantly more isolated than participants with other ‘personality disorders’ (*p* = .002) over a 20-year follow-up period [92]. The prevalence of social isolation did not change significantly over time (odds ratio 0.91, 95% CI: 0.74, 0.14, *p* = 0.42) [92].

Six studies, five of which are longitudinal studies, in this category found a significant relationship between satisfaction and perceived quality of relationships and a ‘EUPD’ diagnosis/traits. People with ‘EUPD’ reported significantly less perceived satisfaction with, and reduced positive perceptions of, their social networks, compared to the general population [29, 36, 40–43]. In one US study sampling female undergraduate students, rated as high-quality, lower perceived satisfaction and quality of relationships over time (1 month) were also associated with more symptoms of ‘EUPD’ [40]. However, in another U.S study consisting of people with a diagnosis of ‘EUPD’, rated as high-quality, increase and exacerbations in ‘EUPD’ traits were associated with changes and reductions in their perception of quality (i.e. support, closeness and satisfaction) with their most frequently interacted with partners within their social network [40].

A cross-sectional study of Dutch and Australian subjects used genetic data from two large samples of twin data, rated as high-quality, found that all traits characteristic of ‘EUPD’, such as affect instability, self-harm, and identity disturbances, were significantly correlated with loneliness, with identity disturbances being most highly correlated [91]. Two studies, one of which was rated as high-quality, found that levels of loneliness and relationship quality were significantly associated with ‘EUPD’ symptoms even when other relevant social and psychological variables were controlled for, such as trauma and baseline relationship quality [87, 88].

### PSS and ‘Emotionally Unstable Personality Disorder’ diagnosis or traits

Nine cross-sectional studies in this category assessed PSS among people with traits or a diagnosis of ‘EUPD’, of which one study was a randomized controlled trial (RCT) (See Supplementary Table 9). Five US studies, three of which are rated as high quality [45, 93, 94], indicated that people with higher numbers of traits associated with ‘EUPD’ reported low levels of PSS [45, 93, 94], lower satisfaction with social support networks (-0.21, *p* < 0.01) [95], and lower emotional support compared to the general population [29]. One US study rated as high-quality reported a correlation coefficient of –0.36 (*p* = .000) [45]. Another US study rated as high-quality found that greater social support was associated with lower ‘EUPD’ traits and that social support was significantly inversely associated with 10 ‘EUPD’ symptoms, including interpersonal problems/distrust and emptiness [94].

## Discussion

We found a total of 70 (*n* = 55 cross-sectional studies) studies that reported on the prevalence and/or severity of loneliness and deficits in PSS among people with a diagnosis/traits of ‘personality disorder’. These provided substantial evidence to support a positive association between a diagnosis/traits of ‘personality disorder’ and loneliness and deficits in PSS. People with a diagnosis/traits of ‘personality disorder’ reported higher levels of loneliness in comparison to other clinical groups (i.e. depression, psychosis) and the general population. The results of this review indicated that people with a diagnosis/traits of ‘EUPD’ and ‘AVPD’ consistently experienced higher levels of loneliness and deficits in PSS. The findings also point to a positive association between schizotypal/schizoid/paranoid ‘personality disorder’ traits/diagnosis and loneliness/PSS. However, for narcissistic traits the findings suggest that there is a complex relationship between these and loneliness/PSS, which is specific to individual narcissistic dimensions (vulnerable/covert narcissism and grandiose/overt narcissism). We also found weak evidence from longitudinal studies that greater reported loneliness and low satisfaction with social network are associated with a greater number of ‘personality disorder’ traits over time, but this must be interpreted in the context of poor study quality. The certainty of evidence for the relationship between all types of ‘personality disorders’ and loneliness and PSS was judged to be low (see Supplementary Table 10).

### Findings in the context of other studies

There are no clear comparator reviews, as this was the first systematic review of quantitative studies examining loneliness and deficits in PSS among people with ‘personality disorder’ diagnoses/traits. In light of the importance of subjective social concepts and to promote comprehensiveness of the review, we had a broad inclusion criterion for social concepts and included any study that reported on subjective social concept. The decision to include a wide range of social concept was based on Wang et al’s [26] proposed conceptual model which maps and categorises the social concepts that encompass and relate to social isolation. Our findings can be interpreted in the context of two qualitative meta-syntheses, one of the studies exploring what service users with diagnosis of ‘EUPD’ view as important for recovery [96] and another summarising qualitative literature on the experience of loneliness among people with ‘personality disorders’ [1], which emphasised that social factors, particularly social disconnection and poor social support, are a prominent concern among people with ‘personality disorder’ diagnoses/traits [1, 96].. Indeed, people with ‘personality disorder’ diagnoses/traits perceive that these factors are linked to suicidal thoughts, suicidal ideation, and suicidal behaviour [1]. A previous mixed-methods systematic review that explored chronic emptiness among people with a diagnosis/trait of ‘EUPD’ demonstrated that feelings of emptiness, which reflects a sense of detachment from others, is strongly associated with impulsive behaviours such as self-harm and suicide attempts [97].

Quantitative studies, qualitative studies and theoretical models on loneliness suggest that people with symptoms of ‘personality disorder’ who are also lonely have difficulties establishing a sense of belongingness, which may further contribute to heightened hypervigilance rooted in painful rejecting childhood and adult experiences [1, 98, 99]. This systematic review has demonstrated that people with ‘EUPD’ and ‘AVPD’ experience higher levels of loneliness and deficits in PSS compared to other types of ‘personality disorder’. This may be due to deficits in social cognition that are thought to be central in ‘personality disorder’, particularly among people with ‘EUPD’ [100]. People with a ‘EUPD’ diagnosis have been found to be sensitive to subtle facial cues of rejection and threat [101], appraise neutral faces as less trustworthy [102], and show high emotional contagion [103]. People with ‘NPD’ show deficits in identifying facial emotions and social cognitive abilities [104]. Such social cognitive difficulties and consequent perceptions appear to exacerbate symptoms of ‘personality disorder’ such as difficulties emotionally regulating, feelings of emptiness, increased impulsivity, and increased psychological distress via further reinforcing negative self-perceptions and self-esteem [1, 98, 105]. Importantly, social cognitive functioning is also linked to childhood attachment insecurity, a reported chronic issue among people with a ‘personality disorder’ [106]. Based on our previous meta-synthesis on loneliness among people with ‘personality disorder’ diagnosis/traits, these social cognitive working models shaped by childhood attachment styles and experiences probably plays a role in the bi-directional relationship between loneliness an ‘personality disorder’ symptoms.

Quantitative studies have also supported the potential to promote a sense of belonging and reduce loneliness in those with ‘personality disorder’ diagnoses/traits as a means of improving self-harm outcomes and valued personal recovery outcomes [107, 108]. A systematic review of longitudinal studies examining the relationship between loneliness/PSS and mental health found that loneliness/PSS predicted poorer depression outcomes in terms of recovery and symptoms [6]. The findings from that review suggests that this might be potentially true for ‘personality disorder’ diagnoses/traits, however it is important to emphasise that there is a lack of longitudinal evidence from our current review to support causality in relation to ‘personality disorder’.

Other studies in the field of PTSD have also established that trauma and intense feelings of loneliness are associated with suicidal ideation, emotional dysregulation, feelings of emptiness and impulsivity [98]. Previous work has shown that often a history of trauma, which has been posited as the core issue for people with diagnoses/traits of ‘personality disorder’, along with intense feelings of alienation and loneliness is associated to poor mental health and suicidal ideation [98].

### Strengths and limitations

We conducted the first systematic review of quantitative studies examining the prevalence and severity of loneliness and deficits in PSS among people with a diagnosis/traits of ‘personality disorder’. Importantly, this is the first systematic review that includes a range of methods used to measure loneliness and PSS among people with diagnoses/ traits of ‘personality disorder’. It is also the first review to contrast subjective social factors among people with ‘personality disorder’ diagnoses/traits to those for other clinical groups and the general population. The definition of concepts, the selection of search terms, and the inclusion criteria were discussed thoroughly with a wider multidisciplinary team of senior researchers, clinicians, and people with relevant lived experience to ensure a comprehensive set of varied search terms. We gained lived experience input into the formulation of our research question, design of our search strategy, inclusion/exclusion criteria, search strategy, review protocol, and reporting of findings. We pre-registered the study protocol on PROSPERO for transparency.

One limitation of this review relates to our search strategy, which despite the efforts outlined above, may not have retrieved some articles. Upon conducting a Google search, 18 published studies were missed in our initial search of the four databases, and were included after eligibility screening, demonstrating the importance of this aspect of the search strategy. As most studies identified in our search were cross-sectional, we were not able to identify the direction of causation in the associations reported. However, research in depression and anxiety support the idea that mental health symptoms contribute to loneliness and vice versa; and that the relationship is likely bidirectional, with psychological and social factors influencing these pathways [1, 4].

As our aim was to be comprehensive and inclusive of all types of ‘personality disorder’ traits and diagnoses, the different forms of diagnostic measures used to assess ‘personality disorder’ traits and diagnoses (and their varying validity) limits the generalizability of our findings to all those with ‘personality disorder’ traits and diagnoses. It is important to note that the majority of studies identified focussed on ‘Cluster B personality traits’, primarily ‘EUPD’ traits, as well as on women and participants from high income countries, which also limits generalizability of our findings. The focus on gender disproportion may be attributed sampling bias, higher likelihood of women in mental health settings and clinical bias in diagnosis [109]. In taking a broad approach to our inclusion criteria, we identified studies with a range of aims and methodological approaches, including social network analyses and cross-sectional questionnaire studies. This heterogeneity of assessment measures and methods, along with the different study designs and varying population groups and settings, and the consequent reliance on a narrative synthesis to report findings, hinders the ability to formulate clear and specific conclusions. It also impacted our ability to compare loneliness levels across studies and conduct a meta-analysis.

### Clinical, policy and research implications

There is a lack of robust longitudinal studies in this field and a need for more longitudinal studies with longer-term follow-up to clarify the relationship between loneliness/PSS and recovery and symptomatic outcomes among people with ‘personality disorder’ diagnoses/traits; based on our findings, currently the extent to which they are causally related is unclear. There is a need to cautious in interpreting our findings and implications. Including standardised and validated loneliness measures and ‘personality disorder’ measures in longitudinal datasets, with a focus on strategies to address incomplete follow-up, will be important pre-requisites for investigating these pathways and establishing direction of causality. Furthermore, there is lack of research investigating the mechanisms by which loneliness may exacerbate ‘personality disorder’ and how psychological symptoms and environmental factors play into the relationship between loneliness and ‘personality disorder’. It would be useful to pinpoint the specific traits of ‘personality disorder’ that may increase loneliness and are barriers to maintaining social connections. Additionally, it is important to explore the experience of loneliness in people with diagnoses/traits of ‘personality disorder’ from neurodiverse groups given the growing evidence of its co-occurrence [110]. It is also essential that clinicians assess for social issues such as loneliness with people who may have a diagnosis or traits of ‘personality disorder’. Pending the findings of future research on the effectiveness of socially focused interventions targeting loneliness, clinical efforts to facilitate a sense of belongingness could potentially improve recovery outcomes and reduce self-harm and suicidality [88]. This could involve exploring social cognitions, including the complex links between affective symptoms and psychological factors and feelings of loneliness, and supporting service users in creating and maintaining meaningful social relationships [111]. A movement towards raising awareness among both clinicians and service users on the ways in which loneliness and lack of PSS is associated with recovery outcomes would motivate and promote open conversations in the clinical setting about one’s sense of belonging and its influence on mental health. A focus on relational aspects could contribute to intervention development to target loneliness by facilitating a sense of belonging, both in the therapeutic alliance and in day-to-day life and recovery, as opposed to a focus predominantly on non-suicidal self-harm.

In light of the prevalence of loneliness and deficits in PSS and previous qualitative research demonstrating that people with ‘personality disorder’ diagnoses express a need for a holistic therapeutic approach particularly targeting their unmet social needs, a co-produced psychosocial intervention that targets loneliness and promotes a sense of belonging among people with ‘personality disorder’ diagnoses/traits is a priority [1, 15]. It has been proposed that relational practice and theories that incorporate personal, relational and social factors should shape the care people with ‘personality disorder’ diagnosis/traits receive [15]. Indeed, our findings highlighting the severity of loneliness and deficits of perceived social support strongly supports the need to incorporate relational and social factors into treatment. This is particularly important given that service users, people with relevant lived experience and professionals have called for the improvement of quality of care and broadening of interventions offered for people with ‘personality disorder’ diagnoses/traits to include unmet needs expressed [2, 13, 15]. Recent strategies for loneliness used by the Community Navigator Study, developed for people with overlapping difficulties such as complex depression, and the Groups4Health intervention, developed for people with psychological distress, demonstrate promising evidence of effectiveness and acceptability [23, 112]. To be acceptable where adapted and trialled in people with ‘personality disorder’ diagnoses/traits, these interventions would need to be rooted in an understanding of the issues specific to this group, such as negative and discriminatory experiences associated with the diagnosis of ‘personality disorder’ and traumatic experiences faced by people with ‘personality disorder’ diagnoses/traits that exacerbate feelings of loneliness. This is particularly important given that higher rates of perceived discrimination and internalized stigma are associated with loneliness among people with ‘personality disorder’ diagnoses/traits [22]. Further research should investigate whether a targeted social intervention as opposed to a general psychosocial intervention is more beneficial for people with ‘personality disorder’ diagnoses/traits by investigating acceptability and effectiveness.

## Conclusion

This systematic review of 70 studies demonstrated that loneliness and PSS are each associated with ‘personality disorder’ symptoms/traits and diagnoses, except for narcissistic traits. A diagnosis or traits of ‘personality disorder’ are associated with higher levels of loneliness and lower levels of PSS. However, due to the cross-sectional nature of most studies, and quality of evidence judged to be low, this requires further investigation. Given that loneliness is associated with the severity of ‘personality disorder’ and is associated with recovery outcomes, it is important to address loneliness as an intervention target priority and to develop a set of acceptable and effective interventions targeting loneliness among people with ‘personality disorder’ diagnoses/traits.

### Supplementary Information

**Additional file 1: Appendix 1.** Search strategy.

**Additional file 2: Supplementary Table 1.** Inclusion and Exclusion Criteria for studies.

**Additional file 3: Supplementary Table 2.** Table presenting characteristic and quality appraisal of eligible articles on loneliness and all types of CEN (mixed samples).**Supplementary Table 3.** Table presenting characteristic and quality appraisal of eligible articles on PSS and all types of CEN (mixed samples).**Supplementary Table 4.** Table presenting characteristic and quality appraisal of eligible articles on loneliness and narcissistic traits. **Supplementary Table 5.** Table presenting characteristic and quality appraisal of eligible articles on PSS and narcissistic traits. **Supplementary Table 6.** Table presenting characteristic and quality appraisal of eligible articles on loneliness and cluster A ‘personality disorder’ or traits. **Supplementary Table 7.** Table presenting characteristic and quality appraisal of eligible articles on PSS and cluster A ‘personality disorder’ or traits. **Supplementary Table 8.** Table presenting characteristic and quality appraisal of eligible articles on loneliness and ‘emotionally unstable personality disorder’ diagnosis or traits. **Supplementary Table 9.** Table presenting characteristic and quality appraisal of eligible articles on PSS and ‘emotionally unstable personality disorder’ diagnosis or traits.

**Additional file 4: Supplementary Table 10.** GRADE scoring criteria for studies describing prevalence and/or severity of loneliness and deficits in PSS among people with a diagnosis or traits of ‘personality disorder’.

**Additional file 5.** Prisma Checklist.

## Data Availability

All data is published and is under the public domain. The datasets used and/or analysed during the current study available from the corresponding author on reasonable request.

## Abbreviations

CEN: Complex Emotional Needs
EUPD: Emotionally unstable personality disorder
AVPD: Avoidant personality disorder
BPD: Borderline personality disorder
DPD: Dependent personality disorder
SAD: Social anxiety disorder
PTSD: Post-traumatic stress disorder
PSS: Perceived Social Support
SD: Standard deviation
n: Number of participants
F: Females
UK: United Kingdom
U.S: United Stated
