# Supplementary material for: The prevalence and severity of loneliness and deficits in perceived social support among who have received a ‘personality disorder’ diagnosis or have relevant traits: a systematic review
Source: BMC Psychiatry. 2024 Jan 3;24:21. doi: 10.1186/s12888-023-05471-8 (PMC10765693; doi:10.1186/s12888-023-05471-8)
Supplement: Supplementary file 1 — Additional file 1: Appendix 1. Search strategy. [file 12888_2023_5471_MOESM1_ESM.docx]

**Appendix 1: Search strategy**

Concept 1:

Personality disorder MeSH terms: Personality disorder (exp)

OR personality disorder* or borderline state* or borderline person* or borderline disorder* or emotionally unstable person* or emotional instab* or emotional dysfunction* or personality dysfunction* or impulsive personality* or histrionic personality* or narcissistic personality* or antisocial personality* or dissocial personality* or paranoid personality* or schizoid personality* or schizotypal personality* or avoidant personality* or anxious personality* or dependent personality* or obsessive compulsive personality* or anankastic personality* or sociopathic personality* or mixed personality disorder* or Cluster A personality* or Cluster B personality* or Cluster C personality* or unspecified personality* or complex trauma* or complex post-traumatic stress disorder or complex post traumatic stress disorder or CPTSD or C-PTSD or self-harm or repeated self-harm or complex emotional needs or personality difficult*

AND

Concept 2: Loneliness

MeSH terms: Lonely (exp) OR

lonel* or social isolat* or emotional isolat* or objective isolat* or subjective isolat* or social network* or social support or social contact* or social relation* or social capita* or alienat* or social interact* or social support* or confiding or confide or social connect* or social inclu* or social exclu* or Social integration* or social tie*

AND

Concept 3:

MeSH terms: Quantitative method (exp)

OR prevalen* or incidence or incident* or intensity or questionnaire* or survey* or epidemiolog* or cross-sectional stud* or cross sectional or quantitative stud* or longitudinal or cohort stud* or prospective stud* or proportion or frequency or associat* or correlat* or risk factor* or predict* or determinant* or indicator* or case control*
