# Supplementary material for: The prevalence and severity of loneliness and deficits in perceived social support among who have received a ‘personality disorder’ diagnosis or have relevant traits: a systematic review
Source: BMC Psychiatry. 2024 Jan 3;24:21. doi: 10.1186/s12888-023-05471-8 (PMC10765693; doi:10.1186/s12888-023-05471-8)
Supplement: Supplementary file 2 — Additional file 2: Supplementary Table 1. Inclusion and Exclusion Criteria for studies. [file 12888_2023_5471_MOESM2_ESM.docx]

*Supplementary Table 1. Inclusion and Exclusion Criteria for studies*

|  | Inclusion Criteria: | Exclusion Criteria |
| --- | --- | --- |
| Type of study | - Experimental, observational studies, prospective and retrospective cohort studies, and cross-sectional studies that quantitatively assessed and reported on the point/period prevalence (i.e. Odds ratio), or/and severity of loneliness (i.e. Mean differences) or/and deficits in PSS. For experimental studies to be included, baseline measures of loneliness or PSS should have been assessed and reported. | - Studies in which the only measure of prevalence or severity of loneliness or deficits in PSS was after an intervention had been delivered. |
|  | - Studies that use quantitative methods to measure loneliness or concepts that are indicative of the perception of a person’s social world and subjective appraisal of social connectedness as opposed to objective measures of social relationships such as social network size. - Studies where people with ‘personality disorder’ diagnosis/traits constitute a proportion of the sample, provided that findings relating specifically to people with ‘personality disorder’ diagnosis/traits are clearly identifiable. | - Reviews, meta-analyses, qualitative studies, or conference abstracts and protocols. |
| Participants | - Participants with a primary diagnosis of, or who endorse traits associated with, a ‘personality disorder’, regardless of whether this is, previously or currently, assessed using a validated ‘personality disorder’ diagnostic method, self-reported diagnosis, or diagnosis made by a mental health professional. This includes studies with samples from a non-clinical population who have been assessed for ‘personality disorder’ symptoms or related traits. We have included people who would not necessarily meet the criteria for the diagnosis but have ‘personality disorder’ related traits. | - Studies focusing solely on objective measures of social relationships or measures of perceptions for a specific relationship such as perception of support from a parent or partner. |
|  | - Participants with psychiatric comorbidities such as depression, anxiety, or ADHD. The decision to include participants with comorbidities is in keeping with clinical reality in which a high proportion of people diagnosed with ‘personality disorder’ also meet criteria for other mental health conditions, including ADHD and Autism Spectrum conditions (28–30). - Individuals of all age-groups and ethnic backgrounds | - Participants with comorbid chronic physical or organic conditions, developmental disorders (except for ADHD and Autism Spectrum conditions as it is a common comorbidity for ‘personality disorder’), and neurocognitive disorders or traumatic brain injuries. With the aim of capturing loneliness among people with diagnosis/traits of ‘personality disorder’ and the psychiatric comorbidities commonly associated with ‘personality disorder’ and to avoid the conflation of loneliness with physical health issues, people with physical or organic conditions |
| Outcome(s) | - Studies measuring and reporting the proportion of participants experiencing loneliness or describing the severity of loneliness or deficits in PSS |  |
| Language | - Studies written in English and Spanish and no restrictions for publication dates. |  |
