# Supplementary material for: The prevalence and severity of loneliness and deficits in perceived social support among who have received a ‘personality disorder’ diagnosis or have relevant traits: a systematic review
Source: BMC Psychiatry. 2024 Jan 3;24:21. doi: 10.1186/s12888-023-05471-8 (PMC10765693; doi:10.1186/s12888-023-05471-8)
Supplement: Supplementary file 3 — Additional file 3: Supplementary Table 2. Table presenting characteristic and quality appraisal of eligible articles on loneliness and all types of CEN (mixed samples).Supplementary Table 3. Table presenting characteristic and quality appraisal of eligible articles on PSS and all types of CEN (mixed samples).Supplementary Table 4. Table presenting characteristic and quality appraisal of eligible articles on loneliness and narcissistic traits. Supplementary Table 5. Table presenting characteristic and quality appraisal of eligible articles on PSS and narcissistic traits. Supplementary Table 6. Table presenting characteristic and quality appraisal of eligible articles on loneliness and cluster A ‘personality disorder’ or traits. Supplementary Table 7. Table presenting characteristic and quality appraisal of eligible articles on PSS and cluster A ‘personality disorder’ or traits. Supplementary Table 8. Table presenting characteristic and quality appraisal of eligible articles on loneliness and ‘emotionally unstable personality disorder’ diagnosis or traits. Supplementary Table 9. Table presenting characteristic and quality appraisal of eligible articles on PSS and ‘emotionally unstable personality disorder’ diagnosis or traits. [file 12888_2023_5471_MOESM3_ESM.docx]

*Supplementary Table.2* Table presenting characteristic and quality appraisal of eligible articles on loneliness and all types of CEN (mixed samples)

| Citation | Study setting and study design | Sample size N, diagnostic measure/types of assessment,  type of personality disorder | Sample characteristics:  Mean age (SD) or Median and age range, gender (female n),  Ethnicity | Loneliness measure | Key findings on severity of loneliness (including estimates expressed quantitatively) | Quality appraisal |
| --- | --- | --- | --- | --- | --- | --- |
| Abrams et al (1997) | New York, USA    Cross-sectional study | N=47    Personality  Disorder Examination (PDE) | F= 63.8% | Multilevel Assessment Instrument (Presence of a satisfying  Relationship) | Some Cluster A (‘paranoid personality disorder’= - 0.348; ‘schizoid personality disorder’ = - 0.501) and B disorders (‘histrionic personality disorder”= - 0.453) were inversely correlated with presence of a satisfying relationship (P<0.05) | 8/8  Low risk of bias |
| Alasmawi et al (2020) | Southwest London and Camden and Islington, UK    Cross-sectional study | N= 192    Primary diagnosis identified through electronic health records | F=107 (56%)    Mean age= 42.4 (11.4, 18-74)    White=67%  Black=19.3%  Mixed race=8.3%  Asian=3.1%  Other= 2.1% | UCLA Loneliness scale (ULS-8) | People with a ‘personality disorder’ diagnosis were the loneliest on average (M = 24.81, SD = 3.73), followed by common mental disorder (M = 23.49, SD = 4.66), and then psychosis (M = 19.69, SD = 4.89). Mean loneliness was 3.8 points (CI 2.22 to 5.38) higher for people with common mental disorder compared to psychosis, and 5.1 points (CI 3.38 to 6.87) higher for people with ‘personality disorder’ compared to psychosis (P < 0.001).  The adjusted mean loneliness score was 3.9 points (CI 2.15 to 5.72) higher in people with common mental disorder compared to psychosis and 5.0 points (CI 2.88 to 7.05) higher for people with ‘personality disorder’ compared to psychosis.       Diagnosis still remained independently associated with loneliness. The adjusted mean loneliness score was 2.4 points higher for people with common mental disorder compared to psychosis and 2.3 points higher for people with ‘personality disorder’ compared to psychosis. The differences in loneliness between diagnostic groups were partially related to the higher rates of perceived discrimination and internalized stigma among people with ‘personality disorder’ | 8/8  Low risk of bias |
| Eades, Segal, & Coolidge (2019) | Colorado, USA    Cross-sectional study | N=102    The Coolidge Axis II Inventory (a dimensional measure of ‘personality disorder’) | F=72.5%    Mean age= 73.26 (5.69, 61-93)    White= 96 (94.1%)  Mixed white and American Indian= 2 (1.9%)  Hispanic= 1 (1.0%) | The Interpersonal Needs Questionnaire-15 (INQ) | For thwarted belongingness, there were large positive correlations with traits associated with ‘personality disorders’ including avoidant, obsessive-compulsive, paranoid, schizoid, and schizotypal traits and moderate positive correlations with ‘personality disorders’ traits of borderline, dependent, and narcissism. ‘Personality disorder’ traits explained a signiﬁcant amount of variance of thwarted belongingness | 6/8  Low risk of bias |
| Galione (2012) | St. Louis Metropolitan area, Washington, USA    Longitudinal study | N=851    Structured Interview for DSM-IV Personality for ‘Borderline Personality Disorder’ and ‘Avoidant Personality Disorder’ | Age range= 55-64 | UCLA – Loneliness scale Revised (20 item) | Loneliness was significantly and positively correlated with both ‘borderline personality disorder’ and ‘avoidant personality disorder’. The correlation between ‘borderline personality disorder’ and loneliness is 0.31 (p<0.001). The correlation between ‘avoidant personality disorder’ and loneliness is 0.21 (p<0.001). | 9/11  Low risk of bias |
| Hengartner et al (2013) | Zurich, Switzerland  Cross-sectional study | N=511    DSM-IV ‘Personality Disorders’ Questionnaire | F=55.5%    Age range= 20-41 | Structured Psychopathological Interview  and Rating of the Social Consequences of Psychological  Disturbances for Epidemiology | ‘Schizoid personality disorder’ negatively related to feeling loneliness (frequently lonely: B= -0.193 (SE=0.087), p=0.027). ‘Borderline personality disorder’ was associated with feeling lonely (frequently lonely: B=0.354 (SE=0.095), p=0.000), and this is considerably higher than those found in other ‘personality disorder’ dimensions such as ‘histrionic personality disorder’ (frequently lonely: B=0.207 (SE=0.085), p=0.015). ‘Dependent personality disorder’ was signiﬁcantly related to feeling lonely (frequently lonely: B=0.256 (SE=0.081). p=0.002) | 7/8  Low risk of bias |
| Kalliponuska & Laitinen | Finland    Cross-sectional study | Mental growth group:  N=30    Control group:  N=20    The Personality Integration Scale in the Tennessee Self-concept Scale | Mental growth group:  F=63%    Control group=  F=83% | UCLA Loneliness scale (20 item) and Differential Loneliness Scale | Lonely people had higher scores on the ‘personality disorder’ scale. The correlation between loneliness (UCLA loneliness scale) and ‘personality disorder’ is 0.18. The correlation between loneliness (differential loneliness scale) and ‘personality disorder’ is 0.41 (p<0.01), higher than loneliness in psychosis and neurosis. | 6/8  Low risk of bias |
| Lesange & Cope (1988) | London, United Kingdom    Cross-sectional study | N=38    Case register diagnosis and psychiatrist diagnosis | ‘Personality disorder’ group:  F= 52%    Mean age= 44.68    Other diagnoses group:  F= 85%    Mean age=56.95 | Social network interview derived from social behaviour schedule | The ‘personality disorder’ group  expressed more dissatisfaction with close social contacts and with acquaintances than did the group with other diagnoses (mean dissatisfaction for the ‘personality disorder’ group was 0.47 SD=0.11 and for the other diagnoses group was 0.30, SD=0.67, p< 0.05) | 5/8  Low risk of bias |
| Overholser (1991) | Cleveland, Ohio, USA    Cross sectional study | N=43    Structured Interview for DSM-III Personality Disorders and Millon Clinical Multiaxial Inventory | No Criteria group:  F=69.2    Mean age= 41.31 (7.30)    Partial Criteria group:   F=45.5%    Mean age= 36.64(10.87)    Full-Criteria group:  F=100%    Mean age= 35.09 (6.92) | UCLA Loneliness scale- Revised | No differences between groups were seen on the UCLA Loneliness Scale.  This suggests that dependency is independent of social supports, and not  simply a reaction to perceived social loss. The mean loneliness scores for the group meeting full criteria for dependency is 53.27 (10.28) compared to those with the partial criteria 50.18 (8.17) and the no criteria group 44.08 (13.34). | 8/8  Low risk of bias |
| Overholser (1992) | Cleveland, Ohio, USA    Longitudinal study | N=375      Dependent personality style scale | F=205 (Mean age=19.83(3.88)    M=170 (Mean age=19.45 (1.56) | UCLA Loneliness scale | The ‘dependent personality’ style scale has significant effect on the UCLA Loneliness scale at time 1 and at time 2 (10 weeks later). Subjects scoring higher on the dependency scale show consistently higher loneliness. | 7/11  High risk of bias |
| Overholser (1996) | Psychiatric inpatient treatment centre and training programs Cleveland, Ohio, USA    Cross-sectional study | N (patient sample)=43    N control group)=66    Dependent Personality Style Scale and the Interpersonal Dependency Inventory | F (patient group)=30 (69.8%)   Mean age (patient group)= 37.09 (8.51)    Patient group:  Caucasian (91.9%)  African American (2.7%) Hispanic (5.4%)    F(control group)=63  (95.5%)  Mean age (control group) = 25.12 (7.28)    Control group:  Caucasian (88.5%)  Hispanic (11.5%) | UCLA Loneliness scale | Dependency scales are significantly associated with higher levels of loneliness in the patient group. The interpersonal dependency inventory, as indicated by the ‘dependent personality’ style scale, is associated with feelings of loneliness. In the control group, interpersonal dependency is associated with feelings of loneliness. | 7/8  Low risk of bias |
| New Antonia et al (2012) | New York, USA    Cross-sectional study | ‘Borderline personality disorder’ group:  N=79    ‘Avoidant personality disorder’ group:  N=39    Control group:  N=76    Interviewed using Structured Clinical Interview for DSM-IV Personality | ‘Borderline personality disorder’ group:  F=67%    Mean age = 33.8 (11.6,18-65)    ‘Avoidant personality disorder’ group:  F= 38%    Mean age= 32.3 (11.4, 19-63)    Control group:    F=63%    Mean age= 30.4 (9.4, 19-55) | Interpersonal Support Evaluation List (ISEL) | The ‘borderline personality’ and ‘avoidant personality’ group scored lower on subscales of ISEL compared to the control group (Mean ISEL belonging subscale for ‘borderline personality’ group= 17.2 VS control group= 25.3 VS “avoidant personality” group= 16.9, p<0.001). | 7/8  Low risk of bias |

*Supplementary Table.3* Table presenting characteristic and quality appraisal of eligible articles on PSS and all types of CEN (mixed samples)

| Citation | Study setting and study design | Sample size N, diagnostic measure/types of assessment,  type of personality disorder | Sample characteristics:  Mean age (SD) or Median and age range, gender (female n),  Ethnicity | PSS measure | Key findings on PSS (including estimates expressed quantitatively) | Quality appraisal |
| --- | --- | --- | --- | --- | --- | --- |
| Fridell, Ojehagen & Traskman-Bendz (1996) | Sweden    Longitudinal study | N= 75    68% of the sample had a diagnosis of ‘personality disorder’ | F=44    Mean age (male group)= 37.8 (10.5)    Mean age (female group)= 42.4 (16.5) | Interview Schedule for Social Interaction (ISSI) | At the index admission subjects who have repeated suicide attempts rated their social network as less satisfying than subjects who have not had repeated suicide attempts. At follow-up the patients again rated their social network. When comparing index and follow-up ratings, an improvement was found for both subjects who had repeated and non-repeated suicide attempts; and this reached significance in the repeated suicide attempt subjects. At follow-up, repeated suicide attempt subjects still rated a less satisfactory network in comparison to non-repeated suicide subjects. | 9/11  Low risk of bias |
| Hummelen et al (2007) | Psychotherapeutic Day Hospitals Norway  Cross-sectional study | N=2,274  N(social phobia)= 85  N(‘AVPD’)= 223  SCID-II for DSM-IV axis II disorders | F=71%  Mean age= 35 (9) | To assess current subjective experience of  social support, the patients were asked to rate three items  on a 5-point scale (1 none and 5 very much) | 64% of people with social phobia felt appreciated by a friend and 53% of people with AVPD felt appreciated by a friend. 76% of people with social phobia felt they had a warm person committed to them and 58% of people with ‘AVPD’ felt they had a warm person committed to them. Sense of belonging for both groups were 29%. | 6/8  Low risk of bias |
| Marques et al (2012) | USA and Canada    Cross-sectional study | N=326    SCID-II Avoidant Personality Disorder Module | ‘Avoidant personality’ group:    F=34%    Mean age= 33.9 (12.47)    White=77%  Black= 5%  Asian= 7%  Native= 0%  Other=11%    No ‘Avoidant personality’ group:    F= 37%    Mean age= 36.21(14.11)    White=63%  Black=9%  Asian=7%  Native=2%  Other=19% | Multidimensional Scale of Perceived Social Support (MSPSS) | Individuals who endorsed a greater number of ‘avoidant personality disorder’ symptoms reported signiﬁcantly lower levels of perceived social support (B=-0.1, SE=0.036. =5.51, p=0.019), after controlling for social anxiety, depression, age, and gender. Among people with ‘avoidant personality disorder’, and people with social anxiety disorder, endorsement of item 7 (Is unusually reluctant to take personal risks or to engage in any new activities because they may prove embarrassing) was associated with less perceived social support | 8/8  Low risk of bias |
| McMahon,Malow, &Jennings (2000) | Miami, Florida, USA    Cross-sectional study | N=141    Millon Clinical Multiaxial  Inventory-II (MCMI-II) | F=0%    Mean age= 40.75 (6.13)    Black=56.4%  White=36.8  Hispanic=6.8% | Perceived social network inventory (PSNI) | Group means for ‘personality disorders’ and clinical symptom syndrome scales of the MCMI-II were in the clinically elevated range. This whole group is characterized as having limited perceived support quality, with a significant negative association between ‘avoidant personality’ and perceived social support quality (Intercorrelations Among Variables = -0.23,p<.01). The intercorrelations between social support and ‘dependent personality’ is 0.03. | 7/8  Low risk of bias |
| Smyth et al (2015) | London, UK    Cross-sectional study | N= 1, 698    Standardised Assessment of Personality Abbreviated Scale | F=66.7%    White=63.5%  Black Caribbean=8.7%  Black African=13.2%  Other=14.7% | Social support indicators ‘perceived emotional support’ | Having weekly contact with family and friends as well as a high level of emotional support was associated with decreased odds of personality dysfunction in the fully adjusted models. The adjusted odds ratio of emotional support is 0.41 (0.25, 0.66) p< 0.001 | 7/8  Low risk of bias |
| Stevenson et al (2011) | Sydney    Cross sectional study | N=238    N(young age-group)=76    N(middle age-group)=57    N(old age-group)=98    67.2% of the sample is diagnosed with a ‘personality disorder’ | F=62.6%    Mean age (young age-group)=  27.49  (6.63,17-40)    Mean age (middle age group)= 52.38(6.81,41-64)    Mean age (old age-group)= 76.23 (7.44,65-99) | Social Support Questionnaire | Satisfaction with social supports was signiﬁcantly  worse in the ‘personality disorder’ groups in all age groups (young age group (t = 2.2, df =72, p< 0.05), middle age group  (t= 3.5, df = 55, p<0.001), old age group (t=2.4, df=102, p<0.05) | 6/8  Low risk of bias |
| Strandholm et al (2017) | Finland    Longitudinal study | N=218    Structured Clinical Interview and Screen (Personality Questionnaire) | F=81.5%    Mean age= 16.40 (1.61) | Perceived Social Support Scale–Revised (PSSS-R), | Higher social support from close friends is associated with a lower amount of ‘personality disorder’ symptoms among ‘avoidant’ and ‘schizoid personality disorder’ symptoms, whereas the opposite association was found between social support from close friends and both ‘histrionic’ and ‘narcissistic personality disorder’ symptoms. Perceived social support at baseline from close friends predicted change in ‘schizotypal’ and ‘narcissistic’ symptoms, a higher level of perceived support associated with a stronger symptom decline in time. Interactions between perceived social support from family at baseline and time were observed on ‘paranoid personality disorder’, where again a higher level of perceived support predicted a stronger decline in symptom scores. Changes in reported social support did not correlate with ‘personality disorder’ symptom change, except for the positive correlation between social support from family and ‘schizotypal personality disorder’ symptom change (0.23,p<0.001). | 8/8  Low risk of bias |
| Trull et al (2010) | USA    Longitudinal study | N= N/A    Alcohol Use Disorder  and Associated Disabilities Interview Schedule for DSM-IV | N/A | Perceived interpersonal social support (12 items) | Those with a diagnosis of ‘personality disorder’ scored significantly lower on interpersonal social support, compared to those without ‘personality disorder’ (Wave 1= difference: 0.17 (95% CI: 0.15– 0.19) (Wave 2: difference: 0.20 (95% CI:0.18– 0.23) | 8/11  High risk of bias |
| Wilberg et al (2008) | Norway    Longitudinal study | N= 1516    SCID-II for DSM-IV for Axis II disorders.  80% of the sample had a ‘personality disorder’ | F=72%    Mean age= 35 (9) | Current subjective experience of social support. | The ‘personality disorder’ (Not otherwise specified)  patients rated themselves better than patients with specific ‘personality disorder’ on 2 of 3 social support variables. Compared to patients both with a single (P<0.01) or multiple specific ‘personality disorder’ (P<0.001), they more often had a friend that appreciated them, whereas regarding sense of belonging to a community they were only better off than those with multiple ‘personality disorders’  (P<0.05). | 6/8 Low risk of bias |
| Wilberg et al (2009) | Norway    Longitudinal study | N=1,023    SCID-II for DSM-IV axis II disorders | F=72%  Mean age= 35 (10) | To assess current subjective experience of  social support, the patients were asked to rate three items  on a 5-point scale (1 none and 5 very much) | Both diagnoses (‘avoidant personality disorder’ and ‘borderline personality disorder’) was associated with a lower level of social support, ‘avoidant personality disorder’ to a somewhat higher degree than  ‘borderline personality disorder’. ‘Paranoid personality disorder’, ‘dependent personality disorder’ and ‘personality disorder not otherwise specified’ contributed also to a variable degree of variance explaining social support. The correlation between ‘avoidant personality disorder’ and feeling appreciated by a friend is 0.135 (p<0.05) and for sense of belonging it is 0.205 (<0.001). The correlation between ‘borderline personality disorder’ and feeling appreciated by a friend is 0.136 (p<0.05). The correlation between ‘paranoid personality disorder’ and a sense of belonging is 0.104 (p<0.05) | 6/8  Low risk of bias |

*Supplementary Table.4* Table presenting characteristic and quality appraisal of eligible articles on loneliness and narcissistic traits

| Citation | Study setting and study design | Sample size N, diagnostic measure/types of assessment,  type of personality disorder | Sample characteristics:  Mean age (SD) or Median and age range, gender (female n),  Ethnicity | Loneliness measure | Key findings on severity of loneliness (including estimates expressed quantitatively) | Quality appraisal |
| --- | --- | --- | --- | --- | --- | --- |
| Carter & Douglass (2018) | York, UK    Cross-sectional study | N=200    N(middle-aged)= 100  N(older aged)=100    Narcissistic pathological inventory | Middle aged group:  F = 53  Mean age = 48.07 years (5.27, 35-55)    Older aged group:  F= 51  Mean age = 70.89 (5.97, 65-85) | UCLA – Loneliness scale (20 Item) | Older-aged participants reported signiﬁcantly lower levels of narcissism and significantly higher levels of loneliness than middle-aged participants (t(198) = 5.09, p < 0.001). Introducing narcissism explained 14.0% of the variance in loneliness, which was signiﬁcant, F(2, 197) = 17.149, p< 0.001. Narcissism was a negatively correlated with loneliness. | 7/8  Low risk of bias |
| Eades, Segal, & Coolidge (2019) | Colorado, USA    Cross-sectional study | N=102    The Coolidge Axis II Inventory (a dimensional measure of ‘personality disorder’) | F=72.5%    Mean age= 73.26 (5.69, 61-93)    White= 96 (94.1%)  Mixed white and American Indian= 2 (1.9%)  Hispanic= 1 (1.0%) | The Interpersonal Needs Questionnaire-15 (INQ) | For thwarted belongingness, there were large positive correlations with traits associated with ‘personality disorders’ including avoidant, obsessive-compulsive, paranoid, schizoid, and schizotypal traits and moderate positive correlations with ‘personality disorders’, Borderline, Dependent, and Narcissistic traits ‘Personality disorder’ traits explained a signiﬁcant amount of variance of thwarted belongingness | 6/8  Low risk of bias |
| Gąsiorowska et al (2021) | Poland    Cross-sectional study | N=662    Narcissistic Admiration and Rivalry Questionnaire and Communal Narcissism Inventory | F=55.5%    Mean age= 45.98 (14.95) | UCLA Loneliness scale (3 item) | Agentic (0.22, p<0.00), antagonistic (0.26, p<0.00), and communal narcissism (0.13, p<0.00) were associated with higher loneliness. | 4/8  High risk of bias |
| Gokcearslan et al (2021) | Turkey    Cross-sectional study | N=500    Narcissistic personality inventory | F=65% | UCLA Loneliness scale | Correlational value between narcissistic traits and loneliness is –0.250 (p=0.01) | 4/8  High risk of bias |
| Joubert (1986) | Cross-sectional study | N=57    Narcissistic Personality Inventory | F= 70% | UCLA Loneliness scale – Revised | Narcissistic scores correlated with loneliness (-0.133). When social interest is held constant, narcissism was correlated -0.299 with loneliness (p<0.10). The relationship between loneliness and narcissism is non-significant | 3/8  High risk of bias |
| Kaely, Woolgar & Hewitt (2021) | Vancouver, Canada    Cross-sectional study | N=120    The Super Brief Pathological Narcissism Inventory | F= 73.3%  Non-binary= 1.7%    Mean age= 20.8    Asian= 43.3%  White= 30.8%  Hispanic= 5%  Middle eartern/Persian= 4.2%  African= 3.3%  South Asian= 2.5%  Multiple ethnicities= 7.5% | UCLA Loneliness scale (3 item) | The correlation between loneliness and vulnerable narcissism is 0.50 (p<0.001). The correlation between loneliness and grandiose narcissism is 0.20 (p<0.05).  Accounting for the five-factor personality traits, vulnerable narcissism was significantly associated with loneliness (t=3.52, p<0.001). | 8/8  Low risk of bias |
| Lamkin & Clifton (2013) | Georgia, USA    Social network analysis study | N=148    The Narcissistic Personality Inventory and Pathological Narcissism Inventory | F= 53%    Mean age= 19.2 (1.5)    White= 86% | Participants rated quality of their relationship with partners frequently interacting with and perceived closeness with the partners. | Controlling for centrality and the interaction between narcissism and centrality, both vulnerable and grandiose narcissism were significantly related to perceiving others as less likable, less kind, less open to experiences, and less conscientious. Vulnerable narcissism is positively related to envying alters and feeling less close to alters | 4/8  High risk of bias |
| Odaci & Celik (2013) | Turkey  Cross-sectional study | N=424    Narcissistic Personality Inventory | F=50.7%    Mean age= 18.69 (0.97) | UCLA Loneliness scale | The correlation between narcissism and loneliness is 0.42 (p<0.001) | 7/8  Low risk of bias |
| Rogoza et al (2018) | Poland  Cross-sectional study | N=314    Narcissistic  Admiration and Rivalry Questionnaire | F= 67.5%    Mean age = 22.00 (2.76, 16-35) | UCLA Loneliness scale | Narcissism explained the most variance in loneliness. In regard to loneliness, admiration is a negative predictor (B=-0.41 (SE=0.04), p<0.01) and vulnerable narcissism is a positive predictor (B= 0.34 (SE=0.07), p<0.01) | 6/8  Low risk of bias |
| Graham et al (2017) | Scotland, UK    Cross-sectional study | N=264  The Narcissistic Personality Inventory Version 3 | F=71.6%    Mean age= 31.65 (13.24, 16-72)    British=78.2% White European= 17.7%   White North American= 2.0% Mixed race=1.4% | UCLA Loneliness scale (version 3) | The correlation between loneliness and narcissism is –0.022 | 7/8  Low risk of bias |
| Sedikides et al (2004): Study 1 | North Carolina, USA    Cross-sectional study | N=149    The Narcissistic Personality Inventory | F=71.8% | Emotional Loneliness Scale (ELS) and UCLA Loneliness scale | Narcissism was inversely related both to UCLA-LS and ESLS ( –.30 and –.29, p<0.001) | 4/8  High risk of bias |
| Sedikides et al (2004): Study 2 | North Carolina, USA    Cross-sectional study | N=81    The Narcissistic Personality Inventory | F=74.1% | Assessed daily level  loneliness | Narcissism was unrelated to daily loneliness (–.13, p< 0.25) | 5/8  High risk of bias |
| Sedikides et al (2004): Study 4 | North Carolina, USA    Cross-sectional study | N=154    The Narcissistic Personality Inventory | F=68.1% | Emotional Loneliness Scale (ELS) and UCLA Loneliness scale | Narcissism was inversely, marginally, related to loneliness (UCLA-LS= –.28, p<0.08; ESLS= –.14, p<0.10) | 5/8  High risk of bias |
| Sedikides et al (2004): Study 5 | North Carolina, USA    Cross-sectional study | N=155    The Narcissistic Personality Inventory | F=84.5%    White=85% | UCLA Loneliness scale | Narcissism was inversely related to loneliness (–.37, p<0.001) | 6/8  Low risk of bias |
| Zhang et al (2014) | China,    Cross-sectional study | N=396    Narcissistic Personality Inventory | F= 50.2%  Missing= 6.3%    Mean age= 15.82 (4.56) | Loneliness and Social Dissatisfaction questionnaire | The correlation between narcissistic traits and loneliness is -0.05 (non-significant). Narcissism ( -0.21, t = -3.81, p < 0.001) had significant effects on loneliness. | 4/8  High risk of bias |

*Supplementary Table.5* Table presenting characteristic and quality appraisal of eligible articles on PSS and narcissistic traits

| Citation | Study setting and study design | Sample size N, diagnostic measure/types of assessment,  type of personality disorder | Sample characteristics:  Mean age (SD) or Median and age range, gender (female n),  Ethnicity | PSS measure | Key findings on PSS (including estimates expressed quantitatively) | Quality appraisal |
| --- | --- | --- | --- | --- | --- | --- |
| Barry, Kauten, & Lui (2014) | Mississippi, USA    Cross-sectional study | N=185 adolescents    Pathological Narcissism Inventory and Narcissistic Personality Inventory for children | F=26    Mean age = 16.95 (0.73, 16-19)    White= 66.8% Black= 31%  Other= 2.2% | Social Support scale | Grandiose narcissism was significantly correlated with higher social support. Vulnerable narcissism was negatively correlated with social support | 6/8  Low risk of bias |
| Gąsiorowska et al (2021) | Poland    Cross-sectional study | N=662    Narcissistic Admiration and Rivalry Questionnaire and Communal Narcissism Inventory | F=55.5%    Mean age= 45.98 (14.95) | UCLA Loneliness scale (3 item) | Antagonistic narcissism (-0.17, p<0.00) is negatively associated to perceived social support.  Communal narcissism is positively associated with perceived social support (0.07), however the association did not reach statistical significance | 4/8  High risk of bias |
| Strandholm et al (2017) | Finland    Cross-sectional study | N=218    Structured Clinical Interview and Screen (Personality Questionnaire) | F=81.5%    Mean age= 16.40 (1.61) | Perceived Social Support Scale–Revised (PSSS-R), | Higher social support from close friends associated with a lower amount of ‘personality disorder’ symptoms among ‘avoidant’ and ‘schizoid  personality disorder’ symptoms, whereas the opposite association was found between social support from close friends and both ‘histrionic’ and ‘narcissistic personality disorder’ symptoms. Perceived social support at baseline from close friends predicted change in ‘schizotypal’ and ‘narcissistic’ symptoms, a higher level of perceived support associated with a stronger symptom decline over time. Interactions between perceived social support from family at baseline and time were observed on ‘paranoid personality disorder’, where a higher level of perceived support predicted a stronger decline in symptom scores. Changes in reported social support did not correlate with ‘personality disorder’ symptom change, except for the positive correlation between social support from family and ‘schizotypal personality disorder’ symptom change (0.23,p<0.001) | 8/8  Low risk of bias |

*Supplementary Table.6* Table presenting characteristic and quality appraisal of eligible articles on loneliness and cluster A ‘personality disorder’ or traits

| Citation | Study setting and study design | Sample size N, diagnostic measure/types of assessment,  type of personality disorder | Sample characteristics:  Mean age (SD) or Median and age range, gender (female n),  Ethnicity | Loneliness measure | Key findings on severity of loneliness (including estimates expressed quantitatively) | Quality appraisal |
| --- | --- | --- | --- | --- | --- | --- |
| Badcock et al (2016) | University of Western Australia and University of Wollongon-g, Australia    Cross-sectional study | N=551    Wisconsin Schizotypy Scales-Brief | F= 69.1%    Mean age= 20.6 (5.53,15-55) | UCLA Loneliness scale (20 items) | There is a strong and significant correlation between social anhedonia and the three loneliness factors (Isolation rho = 0.57, p < 0.01; Relational connectedness rho = 0.56, p < 0.01; Collective connectedness rho = 0.60, p < 0.01). The correlation between perceptual aberration and loneliness factors were small and significant (Isolation rho = 0.27, p < 0.01; Relational connectedness rho = 0.21, p < 0.01; Collective connectedness rho = 0.17, p < 0.01). Once the shared  variance with the general psychopathology factor was removed, none of the correlations between the sub-factors of loneliness and the schizotypy subscales was signiﬁcant | 5/8 High risk of bias |
| Gvion et al (2015) | Tel Aviv, Israel    Cross-sectional study | N=196    The Structured Clinical Interview for DSM–IV (SCID-II) ‘Personality Disorders’ for Schizoid traits | F=72    Mean age= 39.94 (13.34) | UCLA Loneliness scale (20 item) | Schizoid features contributed to loneliness. Schizoid features were found to be important factors associated with loneliness in both suicide attempters and non-attempters | 7/8  Low risk of bias |
| Gvion et al (2014) | Israel    Cross-sectional study | N=196    The Structured Clinical Interview for DSM-IV(SCID- II) ‘Personality Disorders ‘for schizoid traits | F=72    Medically serious suicide attempters:  N=43  F=26 (60.5%)  Mean age= 37.37(13.31)      Medically non-serious suicide attempts:  N=49  F= 15 (30.6%)  Mean age= M = 40.31(13.76)    Psychiatric controls:  N=47  F=14 (29.8)  Mean age= 40.96(14.07)    Healthy controls:  N=57  F= 26 (45.6)  Mean age= 37.28 (12.34) | UCLA Loneliness scale (20 item) | A significant positive correlation was found for schizoid tendencies and loneliness (correlational value of 0.50 (p<0.001) | 7/8  Low risk of bias |
| Le et al (2019) | Louisiana, USA  Cross-sectional study | N=160  Schizotypal Personality Questionnaire – Brief Revised | F= 83%  Mean age= 19.9 (1.5)  White=77% | UCLA Loneliness scale | All three schizotypal subscales (positive, negative and disorganized schizotypal traits) were associated with loneliness (positive r=0.50; negative r= 0.37, disorganized r=0.39) | 8/8  Low risk of bias |
| Lincoln et al (2021) | Cleveland, USA    Cross-sectional study | N=76      Multidimensional Schizotypy  Scale–Brief | F= 55.2%    Mean age= 19.68 (1.38)    White=56.6%  Asian=26.3%  Hispanic/Latino=13.2%  African American/Black= 6.6%  Other=10.5% | UCLA Loneliness scale | A significant positive correlation was found between loneliness and schizotypy. People who reported higher rates of loneliness also reported more schizotypal traits (r = .40, p < .001). Loneliness is significantly associated with scores on the negative subscale (r = .51, p < .001) and the disorganized subscale (r = .31, p = .007), but not the positive subscale (r = .002, p = .990) | 3/8  High risk of bias |
| Slotema et al (2019) | Netherlands    Cross-sectional study | N=60    N(Hallucination group)=37    N(non-hallucination group)=23    Schizotypal Personality Questionnaire (SPQ) and primary diagnosis of “borderline personality disorder” in accordance with the DSM-5 | F=100%    Mean age (hallucination group)= 39 (19–71)    Mean age (non-hallucination group)= 33 (23–60) | The De Jong Gierveld Loneliness Scale (DJGL) | The severity of both schizotypy and loneliness was associated with the severity of hallucinations. Severity of hallucinations, among people with ‘borderline personality disorder’ was signiﬁcantly associated with the severity of the emotional, social, and total score for loneliness (Spearman’s rho and P-values of 0.381, P = 0.003, 0.376, P = 0.003 and 0.409, P = 0.001, respectively) | 8/8  Low risk of bias |
| Wong et al (2021) | London, United Kingdom  (Study recruited participants online from multiple countries)    Longitudinal study | N (time 1)= 1,599  N (time 2)= 774  N (time 3)=586    Schizotypal Personality Questionnaire – Brief (SPQ-B) | F (time 1)= 73.3%  Non-binary= 1.4%    F (time 2)= 76.1  Non-binary= 1.4%    F (time 3)=75.8%  Non-binary= 1.4% | Loneliness Questionnaire (LQ) | The correlation between ‘schizotypal personality disorder’ and loneliness is 0.610. Based on network analysis (nodal analysis), there is a strong connection between negative dimension of schizotypy and loneliness. Individuals with high schizotypal personality traits show strong correlation between factor 2 (interpersonal symptoms) schizotypal traits and loneliness (adjusted p< 0.001). Cognitive-perceptual symptoms of ‘schizotypal personality disorder’ is also correlated with loneliness (0.365) | 9/11  Low risk of bias |

*Supplementary Table.7* Table presenting characteristic and quality appraisal of eligible articles on PSS and cluster A ‘personality disorder’ or traits

| Citation | Study setting and study design | Sample size N, diagnostic measure/types of assessment,  type of personality disorder | Sample characteristics:  Mean age (SD) or Median and age range, gender (female n),  Ethnicity | PSS measure | Key findings on PSS (including estimates expressed quantitatively) | Quality appraisal |
| --- | --- | --- | --- | --- | --- | --- |
| Aghvinian & Sergi (2018) | California, USA    Cross-sectional study | N= 114    Schizotypal Personality Questionnaire - Brief Version (SPQ-B) | Low schizotypy group:  F = 13 (59.1%)  Mean age = 20.73 (3.44)  Caucasian= 4 (18.2%)  African-American= 1  (4.5%)  Hispanic= 14 (63.6%)  Asian American=2(9.1%)  Multiethnic=1 (4.5%)    High schizotypy  F= 72 (78.3%)  Mean age= 19.96 (2.40)  Caucasian= 13 (14.1%)  African-American= 7 (7.6%)  Hispanic= 48 (52.2%)  Asian American=17 (18.5%)  Multiethnic=7 (7.6%) | MOS Social Support Survey (MOS-SSS) | Signiﬁcant group diﬀerences were found between the low schizotypy group (Median=4.39) and high schizotypy group (Median=3.76) on the MOS-SSS, U=615.00, p≤0.00) | 7/8  Low risk of bias |
| Blanchard et al (2011) | USA    Longitudinal study | N= 175    N(social anhedonia group)= 86    N(control group)= 89    International Personality Disorders Examination (IPDE) | Social anhedonia group:  F =57%    White= 38 (44.2)  African American= 40 (46.5)  Hispanic= 6 (7.0)  Asian= 1 (1.2)  Other= 1 (1.2)    Control group:  F = 53.9%    White= 40 (44.9)  African American= 37 (41.6)  Hispanic= 7 (7.9)  Asian= 2 (2.2)  Other= 3 (3.4) | The Interpersonal Support Evaluation List (ISEL) | Perceived social support is significantly negatively correlated with all IPDE scales (range of r values = 0.37 to 0.50; P values <.005) | 8/8  Low risk of bias |
| Horan, Brown & Blanchard (2007) | USA    Cross-sectional study | N=79 | Social anhedonia group:  N=23  Mean age= 20.1 (1.7)  White=52%    Magical ideation group:  N=17  Mean age= 19.1 (1.5)  White=64%    Control group:  N=39  Mean age=19.6  (4.5)  White=49% | Social support questionnaire (short form) | Correlational value between satisfaction with social support and ‘personality disorder’ scores within the social anhedonia group: Schizotypal= -0.18,  Schizoid= -0.05, Paranoid= -0.29  Correlational values between satisfaction with social support and ‘personality disorder’ scores within the magical ideation group:  Schizotypal= -0.20, Schizoid= -0.57 (p<0.05), Paranoid= -0.16    Higher schizoid scores were  associated with less satisfaction with social support, accounting for 34% of the variance | 6/8  Low risk of bias |
| Ripoll et al (2013) | New York, USA    Cross-sectional study | ‘Schizotypal personality disorder’ group:  N=19    Control group:  N=19    Patients met full DSM-IV criteria for ‘schizotypal personality disorder’ using Structured Interview for  DSM-IV Personality Disorders | ‘Schizotypal personality disorder’ group:    F=32%    Mean age= 38.6 (10.4)    Control group:    F= 68%    Mean age= 33.7 (9.1) | Interpersonal Support Evaluation List (ISEL) | Participants with ‘schizotypal personality disorder’ score lower on the belonging (F=51.6, d.f.=1,29, p<0.001), tangible (F=35.6, d.f.=1, 29, p<0.001), subscales of the ISEL, compared to the control group without ‘schizotypal personality disorder’ | 8/8  Low risk of bias |

*Supplementary Table.8* Table presenting characteristic and quality appraisal of eligible articles on loneliness and ‘emotionally unstable personality disorder’ diagnosis or traits

| Citation | Study setting and study design | Sample size N, diagnostic measure/types of assessment,  type of personality disorder | Sample characteristics:  Mean age (SD) or Median and age range, gender (female n),  Ethnicity | Loneliness measure | Key findings on severity of loneliness (including estimates expressed quantitatively) | Quality appraisal |
| --- | --- | --- | --- | --- | --- | --- |
| Beeney et al (2018) | Pittsburg, USA    Network  analysis study | N=142    N (clinical participants)= 72  N (community participants)= 70    McLean Screening  Instrument for Borderline Personality Disorder | Clinical group:  F= 50 (67%)    Mean Age = 43.15 (9.79)    European American= 41 (57%)  African American = 27 (38%)  Biracial= 4 (6%)  Asian= 0      Community group:  F = 47 (63%)    Mean age= 46.56 (10.82)    European American= 40 (47%)  African American= 27 (39%)  Biracial= 2 (3%)  Asian= 1 (1%) | Participants list 30 individuals/alters and rate perceived closeness, perceptions of criticism and practical support, and emotional support on a 4-point scale | Higher ‘borderline personality disorder’ traits are associated with perceptions of less closeness and emotional support and a poorer social network from alters (people within social network) | 5/8  High risk of bias |
| Belford, Kaehler & Birrell (2012) | University of Oregon, USA    Cross-sectional study | N=165    The Borderline Symptom List–23 | F= 64%    Mean age=19.95 (1.92,18-30)    White=78.2%  Asian=7.3%  African America=3.1%  Other=11.4% | The Relational Health Indice (RHI) | The correlation between RHI community subscore and ‘borderline personality’ features is −0.32 (p<0.001). RHI total score signiﬁcantly predicted ‘borderline personality’ scores when trauma level was controlled, (B =−0.2589, t(163)=−2.9773, p = 0.003) | 5/8  High risk of bias |
| Bohus et al (2007) | Freiburg, Germany    Cross- sectional study | N(female group)= 308    N(male group)= 72    Borderline symptoms list (BSL) | Female group:  F= 308  Mean age= 30 (17-56)    Male group:  Mean age= 31 (18-58) | Borderline symptoms list (BSL) | Comparing ‘borderline personality disorder’ with a control group and patients with Axis 1 disorders, people with ‘borderline personality disorder’ show higher mean values for loneliness levels 1.9 (0.89)  (p<0.001). People with ‘borderline personality disorder’ mean value for loneliness pre-treatment is 1.78 (0.81) | 7/8  Low risk of bias |
| Howard, Lazarus, & Cheavens (2021) | Ohio, USA    Longitudinal study, social network analysis | Study 1  N= 126    Study 2  N= 50    The Personality Assessment Inventory–Borderline Features Scale (PAI-BOR) and the Structured Clinical Interview for DSM–IV-Axis II Personality Disorders | Study 1:  F= 100%  Mean age=19.81 (3.78,18-52)    Caucasian= 65.9%  African American= 14.3%  Asian= 11.9% Hispanic=0.8%  Other= 7.1%    Study 2:  F=100%    Mean age=  28.5 (SD = 9, 19-52)    Caucasian= 70%  African American=16%  Asian= 4%  Hispanic= 4%  Other= 6% | Participants rated quality of their relationship with partners frequently interacting with. | Decrease in perceived relationship quality at baselines to follow-up is associated with increase in ‘borderline personality disorder’ features, controlling for baseline ‘personality disorder’ features. Increase in ‘borderline personality disorder’ features are associated with decreases in relationship quality (1 SD increase in PAI-BOR, partner-level relationship  quality decreases by 0.20 SD) | 11/11  Low risk of bias |
| Lazarus (2015) | Dialectical Behavioural clinics, Department of Psychiatry at the Wexner Medical Centre in Ohio, USA    Social network analysis, longitudinal study (3 months) | ‘Borderline personality’ group:  N=21    Control group:  N=21      Personality Assessment Inventory- Borderline Features scale | ‘Borderline personality’ group:  F=100%    Control group:  F=100%    Mean age (all participants)= 27.36 (8.22)    White= 71.43 | Participants rated quality of their relationship with partners frequently interacting with (i.e. satisfaction) | ‘Borderline personality’ group perceived significantly less support from (t(40) = -2.91, p < .001) and satisfaction with (t(40) = -4.18, p < .001) the members of their networks. The ‘borderline personality’ group and control group did not differ on perceived closeness with network members (t(40) = -1.49, p = .14). Compared to the control group, the ‘borderline personality’ group had significantly more variability in their ratings of support (t(40) = 2.57, p = .01) and satisfaction (t(40) = 2.20, p < .01).  The ‘borderline personality’ group did have more variability in closeness with partners over time, (F(1, 303) = 4.30, p = .04, b = -0.17, SE = 0.08) | 11/11  Low risk of bias |
| Lazarus et al (2020) | Ohio, USA    Social network analysis , Longitudinal study | N= 50    ‘Borderline personality disorder’ group:  N=27    Control group:  N=23 | F= 100%    Mean age= 28.52 (8.98)    White= 70% | Participants rated quality of their relationship with partners frequently interacting with (i.e. satisfaction, closeness and support) | The ‘borderline personality’ group reported significantly less support (p<0.01,  d= -0.82) and satisfaction (p<0.001, d= -1.33) than the control group. The two groups did not differ on closeness (p=0.39, d=-0.25) | 10/11  Low risk of bias |
| Lazarus, Southward, Cheavens (2016) | Ohio, USA    Social network analysis, longitudinal study (1 month) | N=127    The Personality Assessment Inventory-Borderline subscale | F= 100%    Mean age= 19.57 (2.50, 18-32)    White=66.1% | Participants rated quality of their relationship with partners frequently interacting with (i.e. satisfaction and support) | ‘Borderline personality’ features predicted lower social network satisfaction and support at one month for those with high ‘rejection sensitivity’, but did not impact satisfaction or support for those with low ‘rejection sensitivity (satisfaction: F(1, 124) = 4.62, b =−.003, p = .03; and support: F(1, 124) = 5.54, b = −.004, p = .02) | 8/11  High risk of bias |
| Lazarus & Cheavens (2017) | Ohio, USA    Social network analysis | N=53    “Borderline personality disorder” group:  N=27    Control group:  N=26 | ‘Borderline personality disorder’ group:    F=100%    Mean age=30.22 (9.59)    White=78%    Control group:    F=100%    Mean age= 26.38 (7.77)    White=54% | Participants rated quality of their relationship with partners frequently interacting with (i.e. satisfaction, closeness and support) | The ‘borderline personality’ group rated network partners as less supportive (p <.001, d=0.93) and indicated  less satisfaction with relationships (p< .001, d=1.42) than the control group. The two groups did not differ on ratings of closeness in relationships (p= .16, d=0.40). ‘Borderline personality’ group had networks that were more variable in terms of satisfaction with relationships (p <0.01, d=1.01). The ‘borderline personality’ group tended to report more variability in ratings of closeness than the control group (p=0.053, d=0.55) | 8/8  Low risk of bias |
| Liebke et al (2017) | Germany    Cross-sectional study | N=80    International Personality Disorder Examination and Borderline Symptoms List | ‘Borderline personality disorder’ group:    F=100%    Mean age= 27.1 (5.6)    Control group:    F=100%    Mean age= 27.0 (6.4) | UCLA Loneliness scale | ‘Borderline personality disorder’ patients reported higher levels of loneliness than the control group (t=-12.5, p<0.001, Cohen’s d=2.728). Differences between groups in loneliness still remained significant even after controlling for social network features and social functioning, F(1, 73)= 20.1, p <0.001, d=1.015, explaining 22% of the variance in loneliness | 7/8  Low bias |
| Sato, Fonagy & Luyten (2020) | London, UK    Cross-sectional study | N=256    Personality Assessment Inventory-Borderline Features Scale. | F=67.2%    Mean age= 23.77 (6.67, 18-52)    Asian=51.6%  White=37.1%  Mixed=5.5%  African/Caribbean=3.9%  Hispanic-1.6%  Other=0.4% | The Need to Belong Scale | “Need to belong” and ‘borderline personality’ features are significantly associated 0.35 (p<0.01) | 5/8  High risk of bias |
| Nenov-Matt et al (2020) | Munich, Germany    Cross-sectional study | ‘Borderline personality disorder’ group:  N=36    Persistent depressive disorder group:  N=34    Control groups for both:  N= 70    Borderline Symptom List (BSL) | ‘Borderline personality disorder’ (‘BPD’) group:  F=53%    Mean age=28.8 (9.2)    Persistent depressive disorder (PDD) group:  F=44%    Mean age=38.2 (12.3) | UCLA Loneliness scale (German adaptation) | Both persistent depressive disorder (PDD) and ‘borderline personality disorder’ patients reported signiﬁcantly higher levels of perceived loneliness than the matched control group (Contrast PDD vs. controlgroupforPDD: t=6.8, p<0.001; Contrast ‘BPD’ vs. controlgroupfor “BPD”: t=11.2, p<0.001) Patients with ‘borderline personality disorder’ reported even more feelings of loneliness than PDD patients (Contrast PDD vs. ‘BPD’: t=2.1, p=0.04) | 8/8  Low risk of bias |
| Richman & Sokolove (1992) | Boston, USA    Cross-sectional study | N=40    ‘Borderline personality disorder’ group:  N=20    ‘Neurotic’ group:  N=20    People in the ‘borderline personality disorder’ group had a score above  90 on the Spitzer Borderline Scale | ‘Borderline personality disorder’ group:   Mean age= 33.30 (9.49)      ‘Neurotic’ group:   Mean age= 31.30 (8.00) | UCLA Loneliness scale | The t test comparison between both diagnostic groups on loneliness= -3.89 (p<0.001)  Only one dependent variable, aloneness, powerfully differentiated the borderline and neurotic groups, (F == 7.95, p < .01) | 8/8  Low risk of bias |
| Slotema et al (2019) | Netherlands    Cross-sectional study | N=60    N(Hallucination group)=37    N(non-hallucination group)=23    Schizotypal Personality Questionnaire (SPQ) and primary diagnosis of “borderline personality disorder” in accordance with the DSM-5 | F=100%    Mean age (hallucination group)= 39 (19–71)    Mean age (non-hallucination group)= 33 (23–60) | The De Jong Gierveld Loneliness Scale (DJGL) | Severity of hallucinations, among people with ‘borderline personality disorder’ was signiﬁcantly associated with the severity of the emotional, social, and total score for loneliness (Spearman’s rho and P-values of 0.381, P = 0.003, 0.376, P = 0.003 and 0.409, P = 0.001, respectively) | 7/8  Low risk of bias |
| Glenn & Klonsky (2013) | North-Eastern USA    Cross-sectional study | N=198    Structured Interview for DSM-IV Personality assessing ‘Borderline Personality Disorder’ | F=74%  Mean age=15.13 (1.38, 12-18)    White= 64%  Hispanic=14%  African American=10%  Mixed/other= 12% | UCLA Loneliness scale (10-item measure) | Both NSSI disorder and ‘borderline personality disorder’. ‘Borderline personality disorder’ also accounted for a unique variance in loneliness scores (p < .001), over and above NSSI | 8/8  Low risk of bias |
| Schermer et al (2020) | Netherlands and Australia    Cross-sectional study | N (Australian sample)= 4820 twins    N (Dutch sample)= 6509 twins    Borderline Features scale from the Personality Assessment Inventory | Mean age (Australian sample)= 28.93( 5.8, 15–40)    Mean age (Dutch sample)=33.71  (12.44, 14-86) | UCLA Loneliness scale-Revised | For each scale, the monozygotic (MZ) correlation was higher than the dizygotic (DZ) correlation, suggesting genetic effects for the ‘borderline personality’ scales and loneliness. All aspects of ‘borderline personality’ were found to have significant positive phenotypic correlations (rph) with loneliness, with the lowest estimate for the subscale self-harm (rph = .17) and the highest estimates for the total score of the ‘borderline personality’ questionnaire and identity disturbance showing rph = .51 and rph = .54, respectively. The proportion of the phenotypic correlation between the total score of the ‘borderline features’ scale and loneliness due to genetic factors was 51%. For the subscales, the proportion of the phenotypic correlation with loneliness due to genetic factors was 54%, 47%, 57% and 60% for affective instability, identity disturbance, negative relationships and self-harm | 8/8  Low risk of bias |
| Pucker et al (2019) | USA    Longitudinal study | N=290    Diagnostic Interview for DSM–III–R Personality Disorders and the Revised Diagnostic Interview for Borderlines | F=77%    Mean age=27 (6.3)    White=87% | Revised Borderline Follow-Up Interview (evaluation of social isolation) | Patients diagnosed with ‘borderline personality disorder’ were significantly more isolated than Axis II comparison subjects over time (p .002) over a 20-year follow-up period. The prevalence of social isolation within each study group did not change significantly over time (odds ratio 0.91, 95% CI: 0.74, 0.14, p=0.42). | 10/11  Low risk of bias |

*Supplementary Table.9* Table presenting characteristic and quality appraisal of eligible articles on PSS and ‘emotionally unstable personality disorder’ diagnosis or traits

| Citation | Study setting and study design | Sample size N, diagnostic measure/types of assessment,  type of personality disorder | Sample characteristics:  Mean age (SD) or Median and age range, gender (female n),  Ethnicity | PSS measure | Key findings on severity of PSS (including estimates expressed quantitatively) | Quality appraisal |
| --- | --- | --- | --- | --- | --- | --- |
| Barros (2016) | Arizona State University,  USA    Cross sectional study | N=396    McLean Screening Instrument for Borderline Personality Disorder | F=58.5%  Non-binary= 0.002%    Mean age= 21 (6.17)    White= 58%  Latino/Hispanic= 20%  Black= 7.5%  Native= 1.8%  Middle Eastern= 1%  Other race= 3.8% | Multidimensional Scale of Perceived Social Support (MSPSS) | ‘Borderline personality’ features and perceived social support had a significant negative relationship (r = -.361, p = .000) | 7/8  Low risk of bias |
| Chan (2004) | Alberta, Canada    Cross-sectional study | ‘Borderline personality disorder’group:  N=36    Control group:  N=49    Diagnostic Interview for Borderlines - Revised | ‘Borderline personality disorder’  group:  F=100%    Mean age= 35.11 (11.05, 19-59)    White= 91.7%  Native/Metis= 5.6%  Other=2.7%    Control group:  F=100%    Mean age= 29.39 (8.62, 20-54)    White=89.8%  Asian=4.1%  Other=6.1% | Interpersonal Support Evaluation List-12 (ISEL-12) | Discriminant function analysis show that social support could each be used to differentiate between women belonging to the ‘borderline personality disorder’ group and control group (r= -0.679) | 8/8  Low risk of bias |
| Chang et al (2021) | New Jersey, USA    Cross-sectional study | N=157    McLean Screening Instrument for borderline personality disorder | F= 156 (60.9%)  Male = 72 (28.1%)  Gender queer or non-conforming= 23 (1.2%)  Transmale= 1 (0.4%)  Transfemale= 1 (0.4%)    Mean age= 27.89 (9.98, 18-64)    White = 157 (12.1%)  Hispanic/Latinx = 31 (9.0%) Asian = 23 (9.0%)  Black or African–American = 23 (4.3%)  Multiracial or a different race = 11 (2.7%)  Native American = 7  (1%) | Multidimensional scale of perceived social support (MSPSS) | Greater social support was significantly associated with lower ‘borderline personality disorder’. Social support was signiﬁcantly, inversely associated with eight of the 10 BPD symptoms (I.e. interpersonal problems, self- injury, impulsivity, emotional instability, intense anger, interpersonal distrust, chronic feelings of emptiness, and identity disturbance) Greater social support was associated with lower levels of these symptoms | 8/8  Low risk of bias |
| Clifton et al (2007) | Outpatient programmes at Western psychiatric institute and clinic, Florida, USA  Cross-sectional study | N (‘borderline personality group)= 11  N(patients without ‘personality disorders’)= 11 | ‘Borderline personality’ groups:  F=10 (91%)  Patients without ‘personality disorder’:  F=9 (82%) | Participants are asked to rate the network’s emotional support and closeness | All participants indicated that central members of the network are perceived as subjectively closer relationships. Participants with no ‘personality disorders’ are more likely to seek advice and emotional support from members in their network | 6/8  Low risk of bias |
| Carter & Douglass (2008) | Randomised control trial | N=30    Borderline Evaluation of  Severity | F= 80%    Mean age= 29.7 (7.7,18-45)    White= 90% | The Social Provision scale | Social support score in this study (59.2 (15.1) were lower than those reported in other studies, suggesting impaired social support in the sample recruited | 10/13  High risk of bias |
| Beeney et al (2018) | Pittsburg, USA    Social Network Analysis study | N=142    N (clinical participants)= 72  N (community participants)= 70    McLean Screening  Instrument for Borderline Personality Disorder | Clinical group:  F= 50 (67%)    Mean Age = 43.15 (9.79)    European American= 41 (57%)  African American = 27 (38%)  Biracial= 4 (6%)  Asian= 0      Community group:  F = 47 (63%)    Mean age= 46.56 (10.82)    European American= 40 (47%)  African American= 27 (39%)  Biracial= 2 (3%)  Asian= 1 (1%) | Participants list 30 individuals/alters and rate perceived closeness, perceptions of criticism and practical support, and emotional support on a 4-point scale | Higher ‘borderline personality disorder’ traits are associated with perceptions of less closeness, trust and emotional support and a poorer social network from alters | 5/8  High risk of bias |
| O’toole, Diddy & Kent (2011) | USA    Cross-sectional study | N=165    Participants involved in DBT therapy and had a diagnosis of ‘borderline personality disorder’ | Mean age= 37.10 (12.04, 18-68)      White= 78%  Black=7%  Hispanic=4%  Other=11% | Multidimensional Scale of Perceived Social Support (MSPSS) | Mean social support among people with ‘borderline personality disorder’ is 4.56 (SD=1.44) | 4/8  High risk of bias |
| Forsythe (2011) | Ohio, USA    Cross-sectional study | N=300    The Personality Assessment Inventory- Borderline Subscale (PAI-BOR) | F= 47.7%    Mean age = 19.73 (3.24)    White=67%  Asian=15%  African American=10.3%  Other=4%  Hispanic=3.7% | Multidimensional Scale of Perceived Social Support (MSPSS) | The PAI-BOR was significantly negatively correlated with the MSPSS. The correlations between the PAI-BOR and the MSPSS subscales were all negative, Family (r = -.26, p < .001), Friends (r = -.16, p < .01), Significant Other (r = -.24, p < .001) | 6/8  Low risk of bias |
| Zielnski & Veilleux (2014) | USA    Cross-sectional study | N=165    McLean Screening Instrument for borderline personality disorder | F=64.2%    Mean age= 19.09 (1.14)    White=80% | Social support questionnaire | Higher ‘borderline personality’ features are associated with lower satisfaction with social support (-0.21, p<0.01). There was a signiﬁcant direct effect of ‘borderline personality’ features on social support satisfaction (direct effect = -0.08, SE = .04, LLCI = -0.15, ULCI = 0.01) | 5/8  High risk of bias |
