# Supplementary material for: The prevalence and severity of loneliness and deficits in perceived social support among who have received a ‘personality disorder’ diagnosis or have relevant traits: a systematic review
Source: BMC Psychiatry. 2024 Jan 3;24:21. doi: 10.1186/s12888-023-05471-8 (PMC10765693; doi:10.1186/s12888-023-05471-8)
Supplement: Supplementary file 4 — Additional file 4: Supplementary Table 10. GRADE scoring criteria for studies describing prevalence and/or severity of loneliness and deficits in PSS among people with a diagnosis or traits of ‘personality disorder’. [file 12888_2023_5471_MOESM4_ESM.docx]

*Supplementary Table.10. GRADE scoring criteria for studies describing prevalence and/or severity of loneliness and deficits in PSS among people with a diagnosis or traits of ‘personality disorder’*

| Outcome | Effect | Methodological limitations of study design | Risk of bias | Inconsistency | Indirectness | Imprecision | Publication bias | Large effect/Dose-response/effect of confounding variables | Rating |
| --- | --- | --- | --- | --- | --- | --- | --- | --- | --- |
| Loneliness | Most studies show a positive association between ‘personality disorder’ traits/diagnosis and severity of loneliness | ${Very Serious (-2)}^{a}$ | ${Very Serious (-2)}^{b}$ | ${Serious (-1)}^{c}$ | ${Serious (-1)}^{d}$ | No  (Rating not downgraded) | No  (Rating not downgraded) | Large effect:  Not upgraded  Dose-response relationship:  Yes (+1)  Effect of confounding variable:  Yes (+1) | ⊕⊕🌕🌕  Low |
| Perceived Social Support | Most studies show an association between ‘personality disorder’ traits/diagnosis and deficits in perceived social support. | ${Very Serious (-2)}^{a}$ | ${Very Serious (-2)}^{b}$ | ${Serious (-1)}^{c}$ | ${Serious (-1)}^{d}$ | No  (Rating not downgraded) | No  (Rating not downgraded) | Large effect:  Not upgraded  Dose-response relationship:  Yes (+1)  Effect of confounding variable:  Yes (+1) | ⊕⊕🌕🌕  Low |

Symbols to describe certainty in evidence: high certainty ⊕⊕⊕⊕, moderate certainty ⊕⊕⊕, low certainty ⊕⊕, and very low certainty ⊕.

Explanations for ratings:

a: Methodological limitations: The majority of studies are observational and cross-sectional studies which downgrades the evidence to ‘low’⊕⊕ due to lack of ability to establish causality.

b: Risk of bias: Very serious risk of bias for both outcomes loneliness and perceived social support across studies due to the majority of studies conducted being cross-sectional studies with small sample size particularly for studies exploring the main research question we are examining.

c: Imprecision: The magnitude of the association between outcomes (loneliness and perceived social support) and ‘personality disorder’ across studies vary. The magnitude of the association between outcomes and narcissistic ‘personality disorder’ traits particularly varied, with some studies showing a non-significant association.

d: Indirectness: The majority of studies do not address the research question of interest for this review, and we had to extract correlational values/relevant outcomes of interest described across studies. There are also a wide variety of measures used, population groups and study settings across studies. Therefore, we judged the studies to have serious indirectness.
